# Hydrodeoxygenation of Oxygenates Derived from Biomass Pyrolysis Using Titanium Dioxide-Supported Cobalt Catalysts

**DOI:** 10.3390/molecules28227468

**Published:** 2023-11-07

**Authors:** Surachet Hongkailers, Adisak Pattiya, Napida Hinchiranan

**Affiliations:** 1 Department of Chemical Technology, Faculty of Science, Chulalongkorn University, 254 Phyathai Road, Bangkok 10330, Thailand; surachet.hkl@gmail.com; 2 Bio-Energy and Renewable Resources Research Unit, Faculty of Engineering, Mahasarakham University, Kamriang, Kantharawichai, Maha Sarakham 44150, Thailand; adisak_pattiya@yahoo.com; 3 Center of Excellence on Petrochemical and Materials Technology (PETROMAT), Chulalongkorn University, 254 Phyathai Road, Bangkok 10330, Thailand; 4 Center of Excellence in Catalysis for Bioenergy and Renewable Chemicals (CBRC), Chulalongkorn University, 254 Phyathai Road, Bangkok 10330, Thailand

**Keywords:** hydrodeoxygenation, cobalt, titanium dioxide, 4-propylguaiacol, lignin valorization, bio-oil

## Abstract

Bio-oil upgrading to produce biofuels and chemicals has become an attractive topic over the past decade. However, the design of cost- and performance-effective catalysts for commercial-scale production remains a challenge. Herein, commercial titania (TiO_2_) was used as the support of cobalt (Co)-based catalysts (Co/TiO_2_) due to its low cost, high availability, and practicability for commercialization in the future. The Co/TiO_2_ catalysts were made with two different forms of TiO_2_ (anatase [TiO_2_–A] and rutile [TiO_2_–R]) and comparatively evaluated in the hydrodeoxygenation (HDO) of 4-propylguaicol (4PG), a lignin-derived model compound. Both Co/TiO_2_ catalysts promoted the HDO of 4PG following a similar pathway, but the Co/TiO_2_–R catalyst exhibited a higher activity in the early stages of the reaction due to the formation of abundant Ti^3+^ species, as detected by X-ray photoelectron spectroscopy (XPS) and hydrogen–temperature programed reduction (H_2_–TPR) analyses. On the other hand, the Co/TiO_2_–A catalyst possessed a higher acidity that enhanced propylcyclohexane production at prolonged reaction times. In terms of reusability, the Co/TiO_2_–A catalyst showed a higher stability (less Co leaching) and reusability compared to Co/TiO_2_–R, as confirmed by transmission electron microscopy (TEM) and inductively coupled plasma optical emission spectroscopy (ICP-OES) analyses. The HDO of the real bio-oil derived from pyrolysis of *Leucaena leucocephala* revealed that the Co/TiO_2_–A catalyst could convert high oxygenated aromatics (methoxyphenols, dimethoxyphenols, and benzenediols) to phenols and enhanced the phenols content, hinting at its potential to produce green chemicals from bio-feedstock.

## 1. Introduction

Due to environmental concerns and the need for sustainable development, as outlined in the Sustainable Development Goals announced by the United Nations in 2015, the current dependency on non-renewable fossil fuels, including petroleum-based feedstocks, should ideally be replaced by renewable resources [1,2,3]. Lignocellulosic biomasses (LCBs), which consist of cellulose, hemicellulose, and lignin, are promising renewable feedstocks for the production of biofuels and bio-based chemicals [4,5,6]. In the past decade, the technology of LCB valorization has focused on the production of bioethanol or value-added chemicals from the cellulose and hemicellulose fractions [7]. At the same time, the lignin portion is mostly discarded in the waste stream of the refinery process and burnt for internal energy [8]. Since the lignin fraction is rich in aromatic compounds, it represents a promising source of feedstock to produce bio-based aromatics [9,10,11]. 

One potential approach for lignin valorization is to depolymerize the lignin polymer into monomers and dimers followed by upgrading via catalytic hydrodeoxygenation (HDO) to yield partially deoxygenated products, such as phenols and alkylphenols, as well as completely deoxygenated products, including alkyl-substituted benzenes and ring-hydrogenated cyclohexanol, cyclohexane, and others [12,13,14,15]. Phenols are valuable chemicals that are used as building blocks in various chemical syntheses, such as agrochemicals, plastics, detergents, and pharmaceuticals [16]; on the other hand, non-oxygenated arenes are used as solvents and processed into monomers for polymer synthesis [17]. Moreover, aromatics and cycloalkanes can be used as additive compounds for bio-jet fuels [18]. 

Toward this end, noble-metal-based catalysts, especially platinum- [19], palladium (Pd)- [20], and ruthenium (Ru)-based ones [21,22], exhibit exceptional performances in the HDO of lignin-derived compounds, as well as aryl ether bond cleavage and ring hydrogenation. However, their high cost and sensitivity to poisoning by sulfur and nitrogen compounds raise concerns on their economic viability for practical uses. Non-noble-metal-based sulfides [23,24,25,26] and nitrides [27] have also been applied in HDO reactions, but they suffer from the leaching of sulfur or nitrogen that causes catalyst deactivation and sulfur contamination in the product streams [24,25]. Metal-oxide-supported non-noble metal catalysts, especially nickel (Ni) [12,28] and cobalt (Co) [29,30], have also been tested. The Ni catalysts possess a high hydrogen activation activity, which favors the promotion of C–C bond breaking and causes methanation and coke formation. On the other hand, Co-based catalysts display a higher activity in HDO reactions via C–O bond cleavage [31,32].

Among various types of supports, titanium dioxide (TiO_2_) has shown a good performance owing to its defect sites binding with oxygen atoms in the substrate to facilitate HDO [33]. In addition, TiO_2_ also induces a strong metal–support interaction [34] that diminishes coke formation [35]. Nevertheless, the different types of TiO_2_ support (anatase [TiO_2_–A] and rutile [TiO_2_–R]) show a different activity in the hydrotreatment process. While TiO_2_–A has been found to deliver a superior performance in the conversion of guaiacol over Pd [36] and gold [37] catalysts compared to those supported by TiO_2_–R, the combination of Ru and TiO_2_–R has been shown to perform better in the hydrogenation of xylose, a phenomenon which was attributed to the high degree of lattice matching between RuO_2_ and the rutile structure [38]. Mixing Ni particles with TiO_2_–A or TiO_2_–R nanoparticles was found to promote guaiacol conversion in different pathways [39]. Among the Co supported by CeO_2_, ZrO_2_, HZSM-5, Al_2_O_3_, SiO_2_, and TiO_2_–R catalysts, the Co/TiO_2_–R catalyst had a high potential for the HDO of eugenol to produce propylcyclohexanol at 200 °C and 10 bar initial hydrogen (H_2_) pressure [40], whereas the HDO of isoeugenol over Co/TiO_2_–A provided the lowest yield of propylcyclohexane (9 mol%) at 300 °C compared to other supports [41]. However, the effect of different types of TiO_2_-supported Co catalysts has not been comparatively evaluated in the HDO of lignin-derived model compounds and real bio-oil. Moreover, the reusability of these Co-based catalysts, one of the important properties for an industrial-scale production, should be evaluated.

To enrich the catalyst toolbox for biofuels and bio-based chemicals production from LCBs, this research used two forms of commercial TiO_2_ as the support for Co-based catalysts because of its low cost and practicability for commercialization in the future. This research was divided into two parts. First, the effect of TiO_2_ types (TiO_2_–A and TiO_2_–R) on the metal–support interaction and catalytic performance of the Co/TiO_2_-based catalyst for the HDO of 4-propyl guaiacol (4PG), a model lignin compound, was explored. The stability and reusability of the Co/TiO_2_–A and Co/TiO_2_–R catalysts were also comparatively investigated. The most suitable TiO_2_ form obtained from the first part was then assessed for the HDO of real bio-oil derived from the pyrolysis of *Leucaena leucocephala* trunk.

## 2. Results and Discussion

### 2.1. Characterization of Catalysts

The precise Co content in the prepared Co/TiO_2_–A and Co/TiO_2_–R catalysts was measured using inductively coupled plasma optical emission spectrometry (ICP-OES). It was found that the amount of Co in the Co/TiO_2_–A and Co/TiO_2_–R catalysts was 4.92 and 4.59 wt%, respectively, which were close to the target quantity. The TiO_2_ supports and Co/TiO_2_ catalysts showed type IV isotherms with a H4 hysteresis loop (Figure S1), typical of materials containing micro- and meso-/macropores [42]. The textural properties of the TiO_2_ and Co/TiO_2_ catalysts are displayed in Table 1. Comparing TiO_2_–A and TiO_2_–R, the Brunauer–Emmett–Teller (BET) surface area (S_BET_) and total pore volume (V_p_) of TiO_2_–A were higher than those of TiO_2_–R, consistent with a previous study [36]. After Co loading, the S_BET_ and V_p_ of the catalysts slightly increased, which was possibly due to the interparticle void created by Co particles or clusters that acted as sites to adsorb N_2_. In contrast, the pore radius of the Co/TiO_2_ catalysts (ca. 1.5 nm) was smaller than that on pure TiO_2_ supports (ca. 1.9 nm), reflecting that the large Co particles on the surface of the support partially blocked the pores [43].

**Figure 1:**
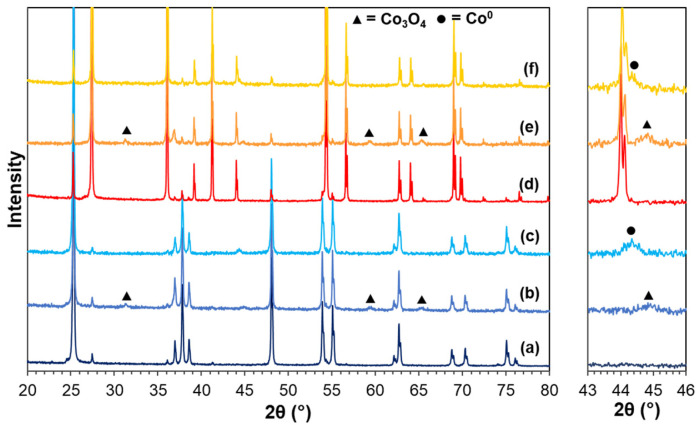
Representative XRD patterns between a 2θ of 20–80° and 43–46° of the (a) TiO_2_–A support, (b) calcined Co/TiO_2_–A, (c) reduced Co/TiO_2_–A, (d) TiO_2_–R support, (e) calcined Co/TiO_2_–R, and (f) reduced Co/TiO_2_–R.

**Figure 2:**
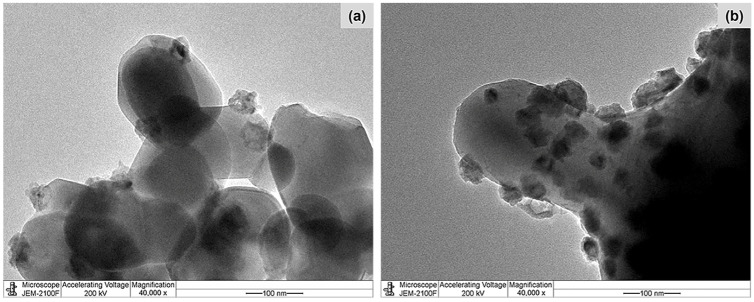
Representative TEM images of the (**a**) reduced Co/TiO_2_–A and (**b**) reduced Co/TiO_2_–R catalysts.

Figure 1 shows the X-ray diffraction (XRD) patterns of the pure supports, calcined Co/TiO_2_, and reduced Co/TiO_2_ catalysts. For TiO_2_–A (Figure 1a), the diffraction peaks at a 2θ of 25.3, 36.9, 37.8, 38.5, 48.1, 53.9, 55.1, 62.1, 62.7, 68.8, 70.2, 75.0, and 76.0° were evident [45], while the XRD pattern of TiO_2_–R (Figure 1d) exhibited 2θ signals at 27.4, 36.0, 39.2, 41.2, 44.0, 54.3, 56.6, 62.7, 64.0, 69.0, and 69.8° [46,47]. After Co loading and calcination, the diffraction peaks of the calcined Co/TiO_2_ catalysts appeared at 31.3, 44.8, 59.5, and 65.4° (Figure 1b,e), which were assigned to Co_3_O_4_ [48]. When the calcined catalysts were reduced, the characteristic peak corresponding to metallic Co^0^ (111) appeared at a 2θ of 44.3° [40] (Figure 1c,f). These XRD patterns indicated that the thermal treatment in the calcination and reduction steps did not cause a phase transformation of anatase to rutile, in accordance with the fact that this typically occurs at a temperature above 600 °C [49]. The metallic Co crystallite size (d_Co_), calculated from the XRD patterns, for Co/TiO_2_–A and Co/TiO_2_–R were 19.1 nm and 27.0 nm, respectively (Table 1). The smaller size of Co in the Co/TiO_2_–A catalyst was explained by the larger surface area of the TiO_2_ support. The size of the Co particles (d_Co_) was larger than that of the TiO_2_ pores, implying that the Co particles were mostly deposited on the outer surface of the supports, as seen in the transmission electron microscopy (TEM) images (Figure 2). Based on the d_Co_ value (Table 1), the metal dispersion (%D_Co_) of Co/TiO_2_–A and Co/TiO_2_–R catalysts was 5% and 3.5%, respectively. This possibly involved the different metal–TiO_2_ interactions, which was further substantiated by the hydrogen–temperature-programmed reduction (H_2_–TPR) analysis.

**Figure 3:**
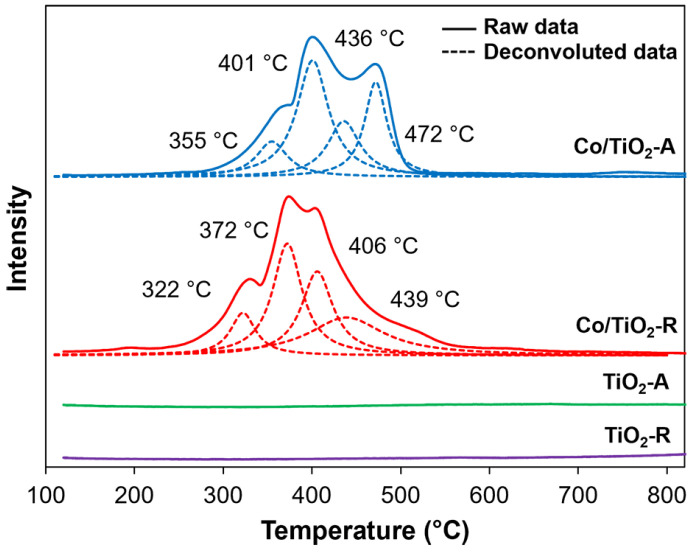
Representative H_2_–TPR profiles of the TiO_2_ supports and Co/TiO_2_ catalysts.

The H_2_–TPR data and H_2_ consumption of the TiO_2_ supports and Co/TiO_2_ catalysts are presented in Figure 3 and Table 2, respectively. The H_2_–TPR profile of the Co/TiO_2_–A catalyst was deconvoluted into four peaks centered at 355, 401, 436, and 472 °C, while the deconvoluted peaks of Co/TiO_2_–R appeared at 322, 372, 406, and 439 °C. The first and second peaks were considered to be the reduction of Co_3_O_4_ to metallic Co [30], consisting of Co_3_O_4_(Co^3+^) → CoO(Co^2+^) and CoO(Co^2+^) → Co^0^ [50]. The third peak was suspected to be the reduction of the TiO_2_ surface. Although the pure supports did not show any peak of H_2_ consumption, it was previously reported that the TiO_2_ surface reduction peak appeared at 340 °C for a Pd/TiO_2_ catalyst [51]. It is reasonable to propose that the addition of Co promoted the reduction of TiO_2_. The final peak was assigned to the reduction of Co species strongly interacting with TiO_2_ [50]. The degree of reducibility was 95.4% and 112.8% for the Co/TiO_2_–A and Co/TiO_2_–R, respectively. The high degree of reducibility of the Co/TiO_2_ catalysts highlights the possibility to reduce TiO_2_. Based on the reduction temperature and the amount of H_2_ consumption, the Co/TiO_2_–R catalyst had a better reducibility than the Co/TiO_2_–A catalyst, indicating the weaker interaction between Co and TiO_2_–R compared to that between Co and TiO_2_–A. 

Next, X-ray photoelectron spectroscopy (XPS) analysis was performed to obtain a better understanding of the chemical compositions of the Co/TiO_2_ catalysts in both the calcined and reduced forms. Deconvoluted spectra of Co 2p obtained from the XPS analysis for the reduced Co/TiO_2_ catalysts are shown in Figure S2. The Co 2p core level XPS signal showed a characteristic 2p doublet separated into 2p_3/2_ and 2p_1/2_ peaks. The first peaks from both the 2p_3/2_ and 2p_1/2_ (at a binding energy [BE] of 778.7 eV and 793.6 eV, respectively) corresponded to metallic Co^0^, while the second peaks of 2p_3/2_ and 2p_1/2_ at a BE of 781.2 eV and 796.3 eV, respectively, were consistent with those observed for CoO_x_ (Co^3+^ and Co^2+^) [30,40]. The last peak, at a BE of about 786.6 eV for 2p_3/2_ and 803.6 eV for 2p_1/2_, was attributed to the satellite peak of CoO_x_ [40]. For the Co/TiO_2_–R catalyst, its XPS spectra showed that peaks appeared at a BE of 778.8, 780.7, and 785.1 eV for 2p_3/2_ and at 794.7, 800.7, and 804.4 eV for 2p_1/2_. The difference in the location of the deconvoluted peaks implied that the Co particles supported by TiO_2_–R and TiO_2_–A had different electronic properties. However, the appearance of the peak related to Co^0^ confirms the presence of active Co in both samples. The Co composition was estimated using the relative peak area and is reported in Table 3. The percentage of Co^0^ was 58.7% and 51.4% in the Co/TiO_2_–A and Co/TiO_2_–R catalysts, respectively, supporting the notion that more Co^0^ species were formed in the Co/TiO_2_–A catalyst than in the Co/TiO_2_–R catalyst. The smaller Co particles of the Co/TiO_2_–A catalyst provided a more uniform reduction, which might be the reason for the higher Co^0^ content in the Co/TiO_2_–A catalyst. 

**Figure 4:**
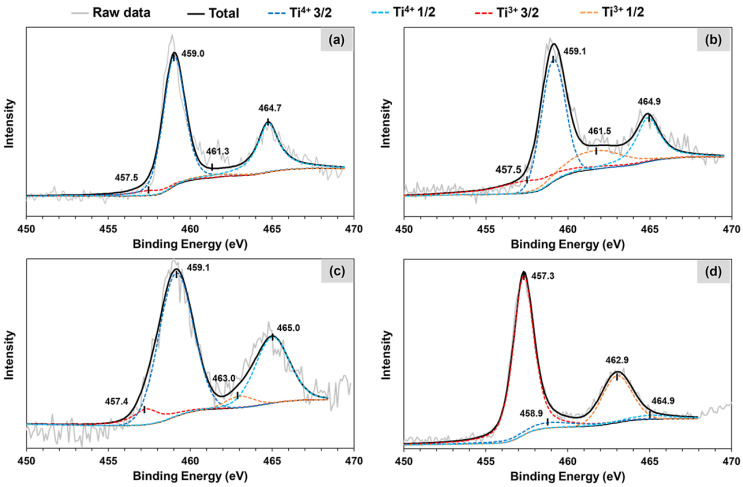
Representative Ti 2p XPS spectra of the (**a**) calcined Co/TiO_2_–A, (**b**) reduced Co/TiO_2_–A, (**c**) calcined Co/TiO_2_–R, and (**d**) reduced Co/TiO_2_–R catalysts.

To further check the surface reduction of TiO_2_ and the oxidation state of Ti, XPS profiles of the calcined and reduced Co/TiO_2_ catalysts were comparatively analyzed, as illustrated in Figure 4, while the composition of Ti species in each catalyst is summarized in Table 3. For the calcined catalysts, the main peak of Ti 2p_3/2_ was observed at a BE of 459.0–459.1 eV in both samples (Figure 4a,c), a phenomenon which was attributed to Ti^4+^ [55,56], while the peak for Ti^3+^ species was observed at a BE of ca. 457.5 eV [57,58]. The percentage of Ti^4+^ was 92.2% and that of the calcined was 88.5% for Co/TiO_2_–A and Co/TiO_2_–R catalysts, respectively. After reduction, the peak at 457.5 eV in the XPS profile of the reduced Co/TiO_2_–A moderately increased (Figure 4b), indicating that H_2_ treatment created a disorder in the TiO_2_ (Ti^4+^) lattice to generate surface Ti^3+^ species [59]. The percentage of Ti^3+^ increased from 7.8% to 26.9% for the reduced Co/TiO_2_–A sample. Surprisingly, the percentage of Ti^3+^ species in the reduced Co/TiO_2_–R catalyst (Figure 4d) was 89.8%, which was three times higher than that in the reduced Co/TiO_2_–A sample. This result was in accordance with the lower reduction temperature of the Co/TiO_2_–R catalyst and the faster surface reduction kinetics of TiO_2_–R compared to TiO_2_–A [60]. In addition, this result was coupled with the lower activation energy for surface reduction of Ru/TiO_2_–R compared with that of Ru/TiO_2_–A, facilitated by H_2_ spillover [61]. Moreover, the formation of Ti^3+^ species at the surface has been reported to be related to the formation of oxygen vacancies (O_v_) [62]. Thus, in this study, the concentration of O_v_ was also calculated using the peak area of Ti species (Ti^3+^ and Ti^4+^) detected by XPS analysis [54], giving an O_v_ concentration of 8.22% and 22.0% for Co/TiO_2_–A and Co/TiO_2_–R, respectively, (Table 3).

The acidity of pure supports and calcined Co/TiO_2_ catalysts was examined using ammonia–temperature-programmed desorption (NH_3_–TPD; Figure S3 and Table 2). The total acidity of TiO_2_–A (45 µmol NH_3_/g) was 1.5 times higher than that of TiO_2_–R (30 µmol NH_3_/g). After Co impregnation, the total acidity increased to 72 µmol NH_3_/g and 46 µmol NH_3_/g for the calcined Co/TiO_2_–A and Co/TiO_2_–R catalysts, respectively, a phenomenon which was due to the additional Lewis acid sites of Co_3_O_4_, as previously reported [63]. The acidity of the reduced Co/TiO_2_–A and Co/TiO_2_–R catalysts decreased due to the transformation of Co_3_O_4_ to metallic Co^0^. However, the total acidity of the reduced Co/TiO_2_–A catalyst (56 µmol NH_3_/g) was still higher (1.8 times) than that of the reduced Co/TiO_2_–R catalyst (31 µmol NH_3_/g).

### 2.2. The HDO of 4PG

#### 2.2.1. Effect of the Reaction Temperature and Reaction Time

**Figure 5:**
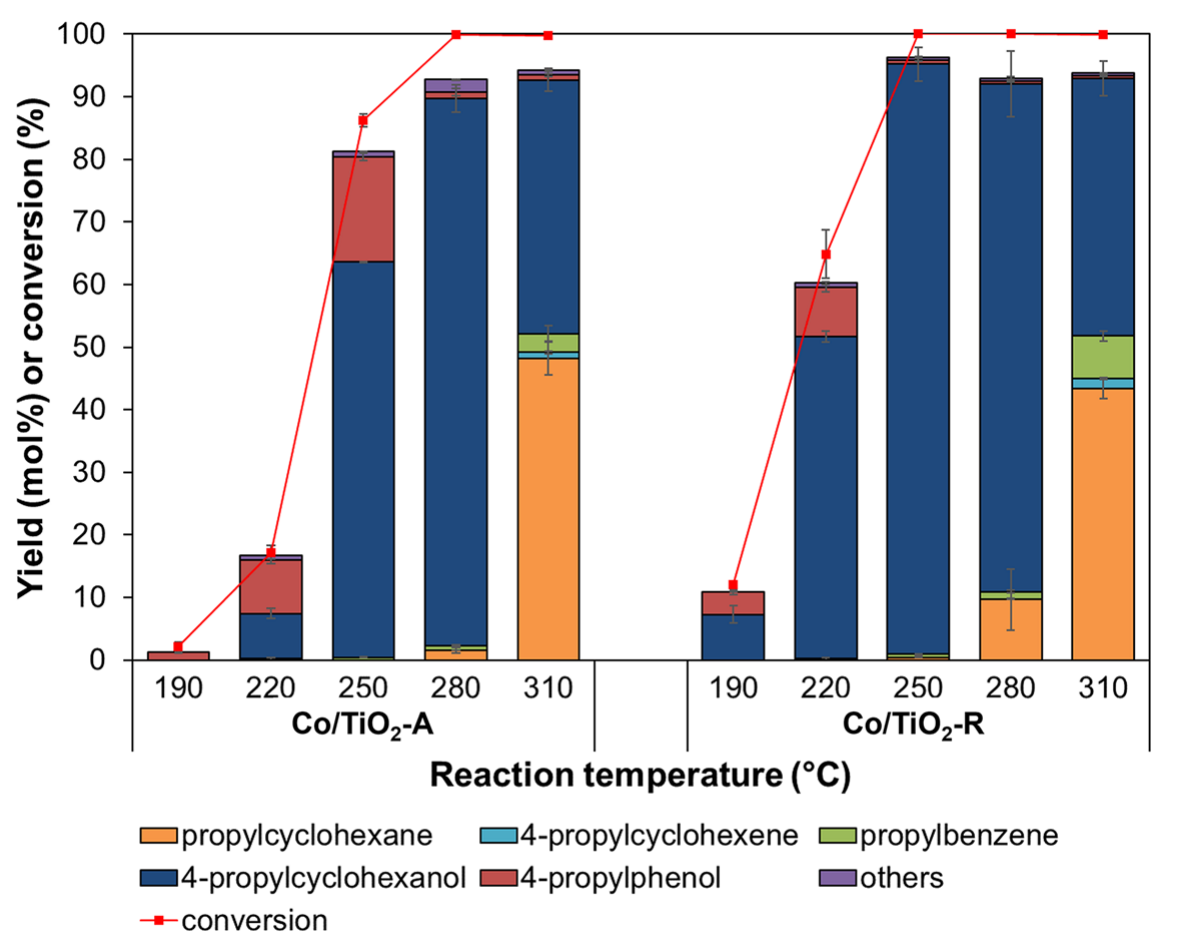
Effect of the reaction temperature on the degree of 4PG conversion and product yields obtained from the HDO of 4PG over the Co/TiO_2_ catalysts (condition: 30 bar initial H_2_ pressure for 1 h).

The catalytic performance of Co/TiO_2_–A and Co/TiO_2_–R in the HDO of 4PG was comparatively investigated under a 30 bar initial H_2_ pressure for 1 h. Liquid products included propylcyclohexane, 4-propylcyclohexene, 4-propylcyclohexanol, propylbenzene, 4-propylphenol, as well as xylenols and alkyl-substituted 4PG. Figure 5 shows the effect of the reaction temperature on 4PG conversion and product yields. For the Co/TiO_2_–A catalyst, a low 4PG conversion level (<20%) was observed when the reaction was performed at 190–220 °C, with the conversion level then remarkably improving to 86.2% at 250 °C and reaching almost 100% at 280–310 °C. Meanwhile, the Co/TiO_2_–R catalyst exhibited a moderate 4PG conversion level (68.7%) at 220 °C and almost complete 4PG conversion (>99%) at 250 °C and above. 

The reaction temperature also affected product selectivity. When the HDO was conducted at 220–250 °C, the Co/TiO_2_–A catalyst formed 4-propylcylohexanol and 4-propylphenol as the dominant products. Increasing the reaction temperature from 250 °C to 280 °C enhanced the 4-propylcyclohexanol yield from 63.2 mol% to 87.4 mol% with a correspondingly decreased yield of 4-propylphenol from 16.7 mol% to 1.0 mol%. This implies that 4-propylphenol was converted to 4-propylcyclohexanol through ring hydrogenation. When the reaction temperature was increased to 310 °C, the product distribution was shifted to 48.3 mol% propylcyclohexane formation with a lower propylcyclohexanol yield of 40.5 mol%, suggesting that propylcyclohexanol was consumed via HDO to produce propylcyclohexane. 

The product distribution obtained from the system using the Co/TiO_2_–R catalyst was similar to that obtained with the Co/TiO_2_–A catalyst. However, the Co/TiO_2_–R catalyst provided a higher yield of 4-propylphenol and 4-propylcyclohexanol at low reaction temperatures (190–220 °C) and a higher yield of 4-propylcyclohexanol (94 mol%) at 250 °C. Increasing the temperature to 280 °C and 310 °C enhanced the yield of propylyclohexane to 9.7 mol% and 43.4 mol%, respectively, at the expense of the yield of 4-propylcyclohexanol which decreased. It should be noted that an observable amount of propylbenzene was obtained at 280–310 °C with both catalysts. It is plausible that the C_aryl_–OH bond was broken via direct HDO at these relatively high temperatures [64]. 

At low reaction temperatures (190–250 °C), the Co/TiO_2_–R catalyst provided a higher 4PG conversion level than the Co/TiO_2_–A catalyst. At 280 °C, the Co/TiO_2_–R catalyst also produced a higher yield of propylcyclohexane, a ring-hydrogenated product. These results imply that the Co/TiO_2_–R catalyst had a higher activity than the Co/TiO_2_–A catalyst to promote the HDO of 4PG. This was attributed to the greater number of O_v_ formed on the surface of TiO_2_–R, as supported by the XPS analysis, which then facilitated C–O bond scission and selective demethoxylation [65]. 

**Figure 6:**
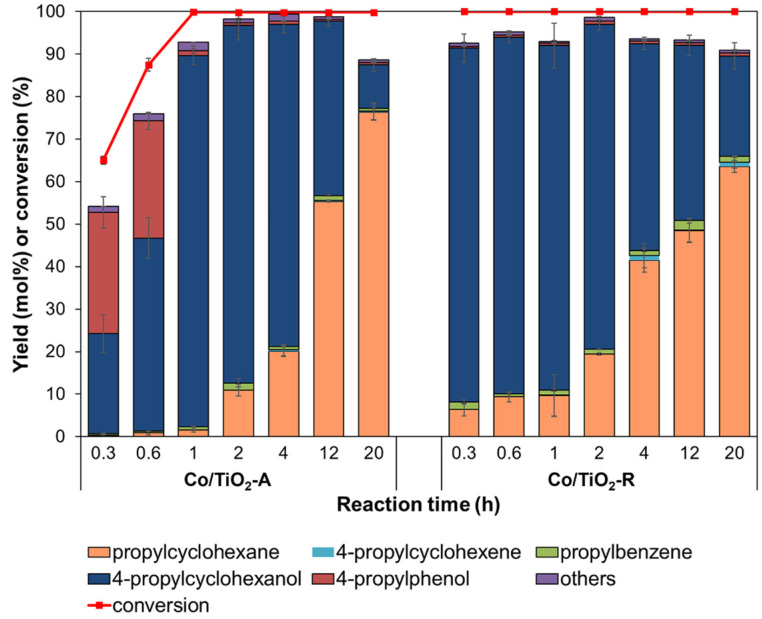
Effect of the reaction time on the degree of 4PG conversion and product yields obtained from the HDO of 4PG over Co/TiO_2_–A and Co/TiO_2_–R (condition: 30 bar initial H_2_ pressure at 280 °C).

The effect of the reaction time on the HDO of 4PG was performed at a 30 bar initial H_2_ pressure and 280 °C (Figure 6). For the Co/TiO_2_–A catalyst, the degree of 4PG conversion was in the range of 65.0–87.5% with the formation of 4-propylcyclohexanol and 4-propylphenol as the main products after 20–40 min. The 4PG conversion level sharply increased to 99.9% within 60 min to form 4-propylcyclohexanol at 87.4 mol% by consuming 4-propylphenol via hydrogenation. As the reaction was prolonged to 20 h, the main product slowly shifted from 4-propylcyclohexanol to propylcyclohexane with a yield of 76.3 mol%, suggesting that HDO of 4-propylcyclohexanol required a long reaction time to obtain the complete deoxygenation.

In contrast, the Co/TiO_2_–R catalyst only gave complete 4PG conversion (~100%) after a 20 min reaction time, with substantial formation of 4-propylcyclohexanol and propylcyclohexane. Although the product distribution was not different from that of the system using the Co/TiO_2_–A catalyst, the Co/TiO_2_–R catalyst affected the sequential reaction for a longer reaction time. When the reaction time was increased from 1 h to 4 h, 4-propylcyclohexanol was predominantly converted to propylcyclohexane, with the maximum propylcyclohexane yield (63.5 mol%) obtained after 20 h. Considering the results from both catalysts, the Co/TiO_2_–R catalyst had a higher ability to promote the HDO of 4PG than the Co/TiO_2_–A catalyst in the early reaction stage. This, possibly, involved the aforementioned greater amount of defect sites on TiO_2_–R to interact with oxygen in the substrate. However, after a prolonged reaction time, the Co/TiO_2_–A catalyst retained its catalytic activity and achieved a higher final propylcyclohexane yield after 20 h than the Co/TiO_2_–R. It was possible that the conversion of 4-propylcyclohexanol to propylcyclohexane occurred through dehydration to produce 4-propylcyclohexene, which could be promoted by the acid sites of the support [66]. Thus, the 1.5-fold higher level of total acid sites of the Co/TiO_2_–A catalyst (as obtained from NH_3_–TPD analysis) might explain the better production of propylcyclohexane from 4-propylctclohexanol by the Co/TiO_2_–A catalyst than the Co/TiO_2_–R catalyst in latter stage of the reaction.

#### 2.2.2. Proposed Reaction Pathway

**Figure 7:**
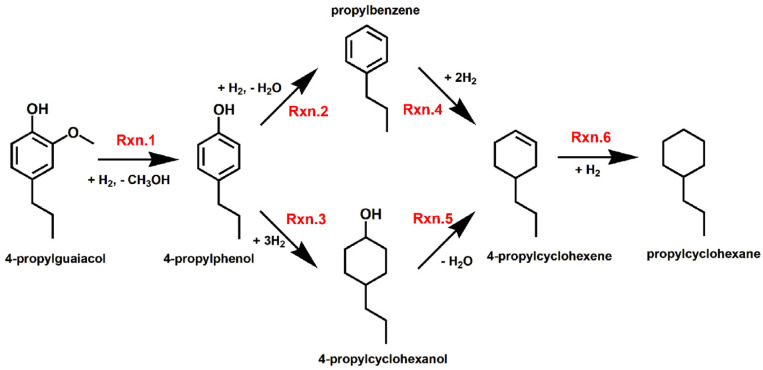
Proposed reaction pathway for the HDO of 4PG over the Co/TiO_2_ catalysts.

Based on the product composition results, the reaction pathway for the HDO of 4PG over the Co/TiO_2_ catalysts is proposed in Figure 7. Note that there are many primary products that could be generated from 4PG conversion, such as 4-propylphenol, 4-propylcatechol, and 1,2-dimethoxy-4-propylbenzene, as well as 2-methoxy-4-propylcyclohexanol [67,68]. However, only 4-propylphenol was observed in this study and 2-methoxy-4-propylcyclohexanol was not detected under any of the tested conditions including a low reaction temperature and short reaction time, implying that 4PG was first converted to 4-propylphenol via demethoxylation (Rxn.1). This can be explained by the metal–support interaction effect of TiO_2_ and Co that created the O_v_ sites to bind with oxygen atoms in the substrates, leading to the selective demethoxylation from among the various other possible reaction pathways [65]. This agrees with an earlier finding that adding TiO_2_ to a Pd/SiO_2_ catalyst improved demethoxylation [69]. Next, 4-propylphenol was transformed into propylbenzene via dehydroxylation (Rxn.2) or into 4-propylcyclohexanol via ring hydrogenation (Rxn.3). Both products were detected, but 4-propylcyclohexanol was dominant, suggesting that the Co nanoparticles catalyzed ring hydrogenation as the major pathway. It has been reported that the degree of hydrogenation depended on the metal particle size [70], a phenomenon which might be explained by the low surface area of the support where catalysts with large metal particles prefer hydrogenation of the benzene ring instead of C_aryl_–OH [71]. 

The obtained 4-propylcyclohexanol and propylbenzene were then simultaneously transformed to 4-propylcyclohexene via dehydration (Rxn.5) and hydrogenation (Rxn.4), respectively. Finally, propylcyclohexane was produced from the hydrogenation of propylcyclohexene (Rxn.6). The proposed reaction pathway over both Co/TiO_2_ catalysts is similar, but there is a difference in terms of the activity of the separate reactions, as seen in the different effects of the reaction temperature and reaction time (Section 2.2.1). 

### 2.3. The HDO of Other Types of Lignin-Derived Model Compounds

To evaluate the feasibility of the Co/TiO_2_ catalysts in the HDO of different lignin-derived model compounds, the HDO of phenol, 4-ally-2-methoxyphenol, and 4-allyl-2,6-dimethoxyphenol, as model compounds of those generally found in depolymerized lignin or lignin pyrolysis oil [72], was tested under a 30 bar initial H_2_ pressure at 310 °C for 1 h. Table S1 shows the conversion level and product yields obtained for each substrate. Both the Co/TiO_2_–A and Co/TiO_2_–R catalysts possessed a high HDO activity for the different model compounds. The major products containing one oxygen atom were generated from partial deoxygenation. Although all the lignin-derived model compounds were completely converted under this condition, the product distribution was different due to the deviation in their number of π-bonds, oxygen atoms, and steric hindrance. For example, structures having more π-bonds, such as 4-ally-2-methoxyphenol, could be hydrogenated, resulting in a lower yield of completely deoxygenated products compared to that from 4PG. Comparing between the Co/TiO_2_–A and Co/TiO_2_–R catalysts, the product distributions obtained from the HDO of 4PG, phenol, 4-ally-2-methoxyphenol, and 4-allyl-2,6-dimethoxyphenol were similar, implying that these two catalysts promoted the reaction following a similar pathway. However, the Co/TiO_2_–R catalyst gave a higher yield of completely deoxygenated products in a short reaction time. This was in line with the result obtained from the HDO of 4PG described in Section 2.2. 

### 2.4. Reusability

**Figure 8:**
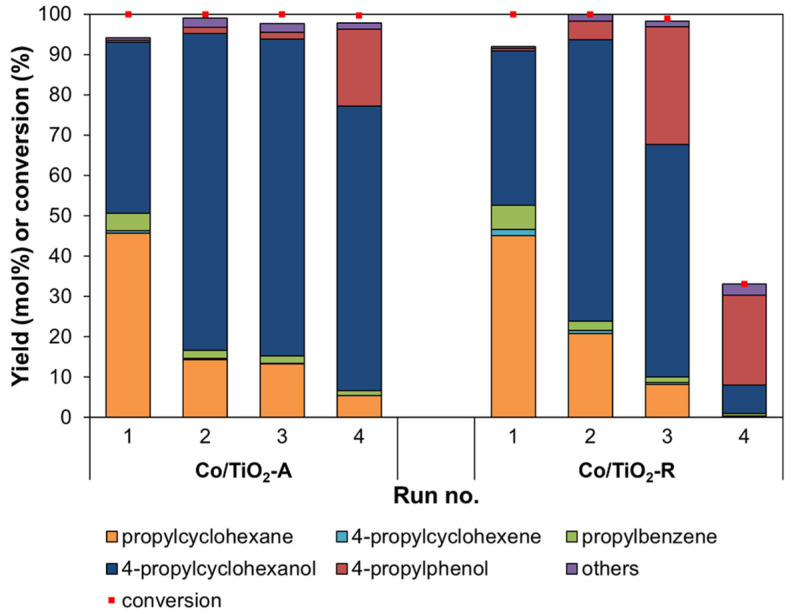
Reusability of the Co/TiO_2_ catalysts obtained from the HDO of 4PG (condition: 30 bar initial H_2_ pressure at 310 °C for 1 h).

To investigate the stability and reusability of the Co/TiO_2_ catalysts, the HDO of 4PG was comparatively tested over four consecutive runs (Figure 8). Based on the results in Section 2.2.1, the reaction was performed at 310 °C, 30 bar initial H_2_ pressure, and 1 h reaction time, since both catalysts showed a similar 4PG conversion level and product distribution under this condition.

It was noticed that the Co/TiO_2_–A catalyst retained 100% 4PG conversion over four runs. However, there were changes in the yield of each product species. The increased 4-propylcyclohexanol and 4-propylphenol yields with reduced propylcyclohexane levels over the four runs indicated the loss of catalytic activity of the Co metal for complete HDO. The 4PG conversion level and product selectivity obtained from the Co/TiO_2_–R catalyst over three runs were similar to those obtained from the Co/TiO_2_–A catalyst; however, the Co/TiO_2_–R catalyst activity significantly decreased in the fourth run, giving only a ca. 30% 4PG conversion level. 

**Figure 9:**
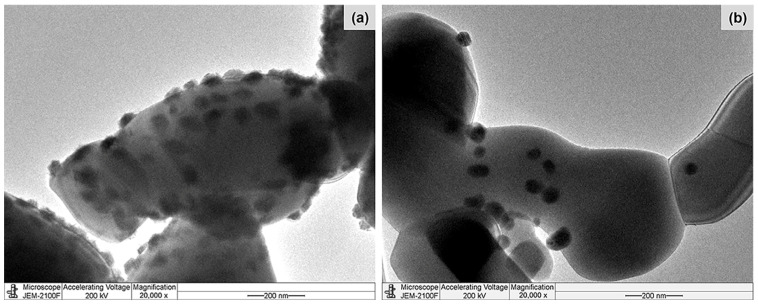
Representative TEM images (20,000× magnification) of the (**a**) fresh and (**b**) spent Co/TiO_2_–R catalysts after the fourth consecutive run.

The degree of coke deposition on the surface of catalysts was determined using thermogravimetric analysis (TGA). This revealed a <1% weight loss (Figure S4), indicating that coke deposition did not cause the deactivation of these catalysts. When the spent catalysts were analyzed after the fourth run using ICP-OES, the Co content in the Co/TiO_2_–A catalyst was found to have slightly decreased from 4.92 wt% to 4.55 wt% (Table S2). However, the amount of Co active metal was remarkably lower for the spent Co/TiO_2_–R catalyst, having decreased from 4.59 wt% to 2.20 wt%. Moreover, the TEM analysis of the spent Co/TiO_2_–R catalyst (Figure 9) also confirmed partial Co leaching. The loss of Co content in the Co/TiO_2_–R catalyst was, therefore, related to its low reduction temperature, implying a deficient interaction between Co and the TiO_2_–R support. According to the product selectivity, it seems that both Co/TiO_2_ catalysts were deactivated. However, the difference in their deactivation was that the Co/TiO_2_–A catalyst could be reused and retained a 100% 4PG conversion level, while the Co/TiO_2_–R catalyst provided only a 30% 4PG conversion level after the fourth run of operation. This was mainly related to the amount of Co leaching.

### 2.5. The HDO of Bio-Oil

The possibility to apply the Co/TiO_2_ catalysts to upgrade bio-oil derived from LCB pyrolysis was also evaluated. In this part, a bio-oil produced from the fast pyrolysis of *L. leucocephala* trunk was used as the raw material for the HDO process. Although the Co/TiO_2_–R catalyst seemed to have a higher HDO activity than that of the model compounds in the short reaction time, the Co/TiO_2_–A catalyst was selected for testing the HDO of bio-oil owing to its stability during long reaction times and its better reusability. Typically, the HDO of the bio-oil in the presence of n-dodecane (0.3 g bio-oil/4 mL n-dodecane) was performed under a 30 bar initial H_2_ pressure at 310–350 °C for 4 h. The comparative distribution of the bio-oil composition and the liquid product obtained from the HDO of the bio-oil is shown in Figure S5. 

The untreated bio-oil contained a 32.4 wt% aqueous phase and a 67.6 wt% organic phase. After HDO at 310 °C for 4 h, an increase in the aqueous phase was observed, which might have resulted from the water generated from the HDO. On the other hand, the organic phase decreased with the formation of solid and gas. When the reaction temperature was increased to 330 °C, the amount of aqueous phase dropped, a phenomenon which was potentially due to promotion of the water gas shift (WGS) reaction over the Co-based catalyst [73]. However, the aqueous phase then increased when the reaction temperature was elevated to 350 °C, possibly because the exothermic nature of the WGS reaction suppressed the consumption of generated water at higher reaction temperatures [74]. Meanwhile, the amount of gas product continually increased with increasing reaction temperatures, indicating the presence of the WGS reaction and/or hydrocracking of bio-oil to convert the compounds in the organic phase to gaseous products. In contrast, the amount of solid product tended to decrease at higher reaction temperatures. This implies that the solid product formed on the surface of the spent catalysts was soft coke, which was mainly generated from the condensation of oxygen-containing intermediates and then decomposed at 258–400 °C [75]. As shown in Figure S6, the spent catalysts obtained from the HDO of bio-oil at different temperatures exhibited a weight loss at 300–400 °C, confirming the presence of soft coke on the surface of catalysts. Thus, the higher reaction temperature converted the oxygen-containing intermediates to other products instead of condensation or repolymerization to form the soft coke.

**Figure 10:**
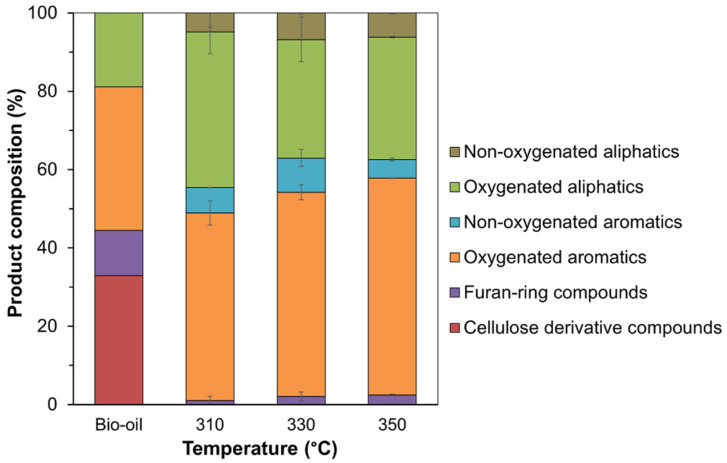
Selectivity to compositions found in the bio-oil before and after HDO over the Co/TiO_2_–A catalyst at different reaction temperatures (condition: 30 bar initial H_2_ pressure for 4 h).

The chemical compositions in the bio-oil before and after HDO were analyzed using gas chromatography mass spectroscopy (GC-MS) and could be classified into six groups according to their structural similarity (GC-MS chromatogram is presented in Figure S7): non-oxygenated aliphatics, oxygenated aliphatics, non-oxygenated aromatics, oxygenated aromatics, furan ring compounds, and cellulose derivative compounds. At the beginning, the bio-oil contained only four groups of compounds: oxygenated aromatics (36.6%), oxygenated aliphatics (18.9%), furan ring compounds (11.5%), and cellulose derivative compounds (33.0%), as presented in Table S3 and Figure 10. When the HDO of bio-oil in *n*-dodecane was performed at 310 °C for 4 h, the cellulose derivatives in the bio-oil totally disappeared, with an increasing content of oxygenated aliphatics (Figure 10). When the reaction temperature was increased to 330 °C and 350 °C, the trend in the product distribution did not significantly change from the results obtained at 310 °C, except for the decreased oxygenated aliphatics and higher oxygenated aromatics formation. It was possible that the oxygenated aliphatics might have been partially converted to gas products. Thus, the gas products obtained from the HDO of bio-oil dissolved in *n*-dodecane at different reaction temperatures (as well as pure dodecane) were also examined by GC equipped with temperature conductivity and flame ionization detectors (GC-TCD/FID). As summarized in Table S4, the concentration of each gas increased with increasing reaction temperatures, implying that the oxygenated aliphatic compounds were converted into gaseous products. 

**Figure 11:**
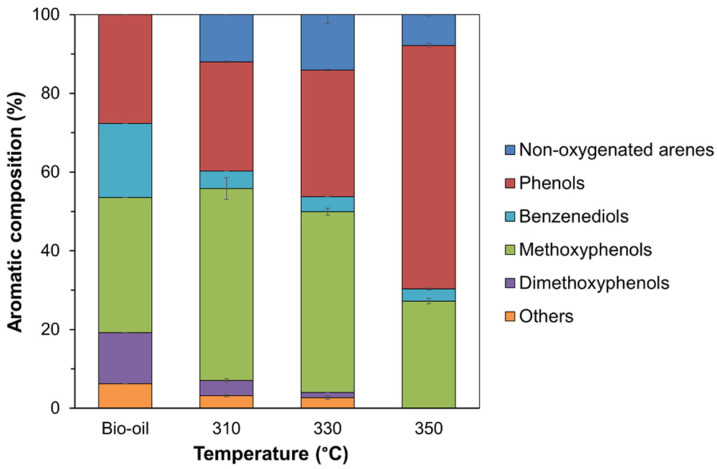
Type and composition of aromatics found in the bio-oil before and after HDO over the Co/TiO_2_–A catalyst at different reaction temperatures (condition: 30 bar initial H_2_ pressure for 4 h).

However, the classification of the product groups as described does not reflect all the changes in the liquid product. For example, 2-methoxyphenol was converted to phenol, but both are categorized as oxygenated aromatics. Considering the type of aromatic compounds, they were divided into six groups: arenes without oxygen atom, phenols, methoxyphenol, dimethoxyphenols, benzenediols, and others. As shown in Table S5 and summarized in Figure 11, the aromatic compounds in the bio-oil consisted of 34.3% methoxyphenols, 27.6% phenols, 18.8% benzenediols, 13.0% dimethoxyphenols, and 6.3% others. 

When the reaction was operated at 310–330 °C under a 30 bar initial H_2_ pressure for 4 h, the content of aromatic compounds containing two or three oxygen atoms, such as methoxyphenols, benzenediols, dimethoxyphenols, and others, continually decreased, while the phenol content increased. In addition, the formation of non-oxygenated arenes was found under these reaction conditions. When the reaction temperature was increased to 350 °C, dimethoxyphenols and others totally disappeared, the content of methoxyphenols decreased to 27.2%, and the content of phenols increased to 61.9%. In terms of gas products (Table S4), a remarkably high C_1_ gaseous concentration was observed when the reaction was conducted at 350 °C. Despite the gas formation from aliphatic moiety, this result shows the possibility of methane formation in the gas phase from the methoxy group.

**Figure 12:**
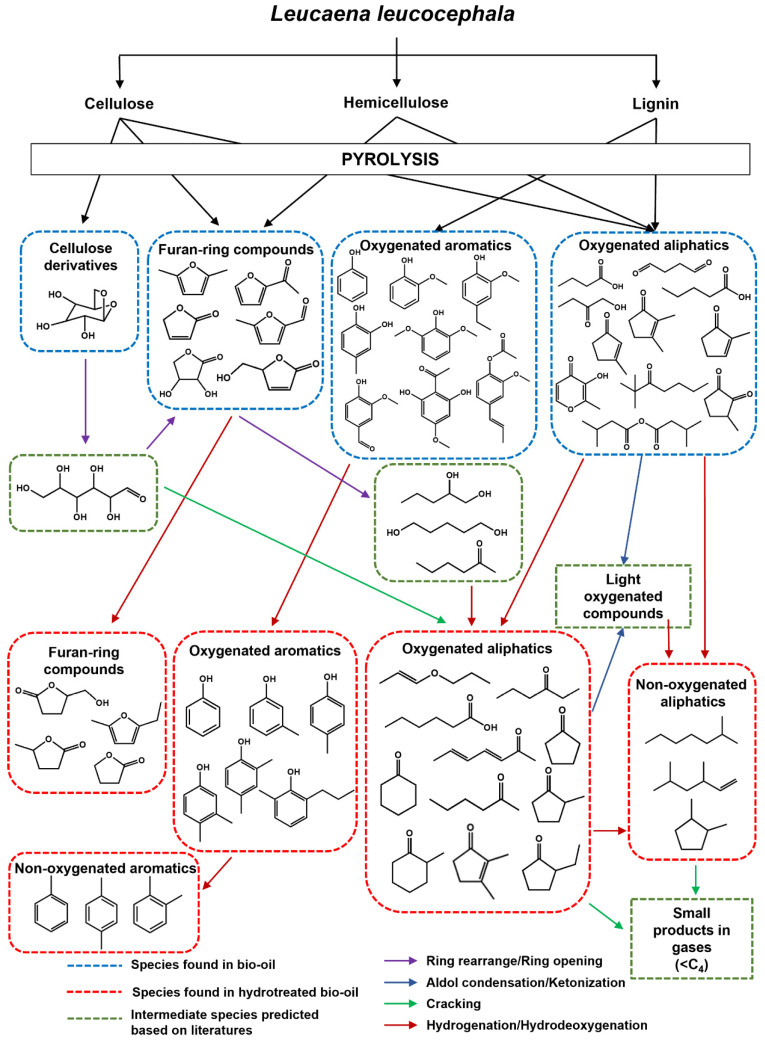
Proposed reaction pathway for the HDO of bio-oil derived from *L. leucocephala* trunk over the Co/TiO_2_–A catalyst.

Based on the compositions found in the bio-oil before and after HDO at different temperatures, the reaction pathway is proposed in Figure 12. During the HDO process, cellulose derivatives in the bio-oil derived from pyrolysis of *L. leucocephala* trunk could be converted via several pathways, such as ring rearrangement of anhydrohexose to form furan ring compounds [76,77] or the ring scission of anhydrohexose or C–C cleavage after ring opening to produce C_2–4_ products with carbon monoxide or methanol as by-products [78,79]. In the case of furan ring compounds derived from hemicellulose and rearranged from levoglucosan, they could be converted through hydrogenation and/or HDO to produce furan ring compounds having a higher degree of saturation or less oxygen atoms [79,80]. Another possibility was that the furan ring was opened to produce C_5_ chains or cyclic hydrocarbons with hydroxyl or carbonyl groups, such as pentanol, pentanediols, and cyclopentanone [64,77]. For aromatics derived from the lignin portion, they could join the aforementioned reaction pathway derived from the lignin-derived model compounds to form phenols, arenes, and hydrogenated products. Non-aromatics containing oxygen atoms were possibly converted via hydrogenation and/or HDO or they might have been cracked to produce small products in the gas phase. However, it should be noted that the oxygenated products, especially aldehydes, ketones, and carboxylic acid, were probably coupled to produce light oxygenated species and other long chain (C_6–9_) products via aldol condensation or ketonization [79]. 

## 3. Materials and Methods

### 3.1. Materials

The 4PG (≥99%, FG), cobalt nitrate hexahydrate (Co(NO_3_)_2_·6H_2_O), TiO_2_–A (99.8%, powder), TiO_2_–R (≥99.9%, powder), *n*-dodecane (≥99%), and *n*-octane (anhydrous, ≥99%) were purchased from Sigma-Aldrich (Singapore). Phenol (>98%) was received from Alfa Aesar. 4-Ally-2-methoxyphenol (99%) and 4-allyl-2,6-dimethoxyphenol (>95%) were from Sigma-Aldrich (Singapore). Ethyl acetate (≥99%, HPLC grade) was obtained from VWR Singapore Pte Ltd. (Singapore). H_2_ (99.999% purity), air, and 5% (*v*/*v*) H_2_ in N_2_ were supplied by Air Liquide Pte Ltd. (Singapore).

### 3.2. Catalyst Preparation

The Co/TiO_2_ catalysts (5 wt% Co) were prepared by wetness impregnation. The TiO_2_ support (1 g) was added to 0.05 M of cobalt nitrate hexahydrate solution (3 mL) under stirring for 5 h at room temperature and then dried in an oven at 50 °C overnight. The resulting product was then calcined in a furnace at 500 °C for 3 h and subsequently reduced ex situ under a H_2_ atmosphere at 420 °C for 2 h.

### 3.3. Catalyst Characterization

The ICP-OES was performed using an ICP spectrometer (Thermo Scientific (Waltham, MA, USA): iCAP 6000 series) to identify the exact Co loading on the catalysts. Before testing, 10 mg of sample was digested in 4 mL aqua regia for 3 d and then diluted in deionized water using a 25 mL volumetric flask. The morphology of the catalysts was also examined using field-emission transmission electron microscopy (FE-TEM; JOEL (Tokyo, Japan) microscope model JEM-2100F).

The XRD patterns of the TiO_2_ supports and Co/TiO_2_ catalysts were obtained using an XRD analyzer (Bruker: D8 Advance). The 2θ was adjusted between 20° and 80° at a step size of 0.02° (0.4 s/step). The d_Co_ was also calculated according to Scherrer’s equation from the characteristic peak of Co^0^ at 44.3° [30]. The %D_Co_ was estimated from the d_Co_ value using Equation (1) by assuming spherical Co particles with a site density (S_d_) of 14.6 atom/nm^2^ [30,44].

(1) $$D_{\mathrm{Co}}\;\left(\%\right)=\frac{6.59\times S_{d}}{d_{\mathrm{Co}}\;(\mathrm{nm})}$$

The N_2_ adsorption–desorption isotherms of the supports and Co/TiO_2_ catalysts were analyzed using a BET analyzer (Quantachrome (Boynton Beach, FL, USA) NOVA touch 4LX) to evaluate the S_BET_, V_p_, and pore radius (r_p_). Each sample (50 mg) was degassed at 300 °C for 3 h before testing. The r_p_ was estimated using the cumulative desorption data derived from the Barret–Joyner–Halenda (BJH) method.

The H_2_–TPR and NH_3_–TPD profiles of the TiO_2_ supports and calcined Co/TiO_2_ catalysts were accomplished using a Belcat-Basic Chemisorption analyzer (BELCAT II). For H_2_–TPR analysis, the samples (50 mg) were pretreated in an argon (Ar) atmosphere (flow rate of 30 mL/min) at 100 °C for 0.5 h before applying 5% (*v*/*v*) H_2_/Ar mixed gas (flow rate of 30 mL/min) for reduction at a ramp rate of 10 °C/min. The degree of reducibility was calculated from the ratio between the total amount of H_2_ consumption obtained in the H_2_–TPR and the theoretical value required to convert Co_3_O_4_ to Co^0^ [52]. The acidity of the samples was evaluated using NH_3_–TPD analysis. After pretreating with helium (He) at 50 mL/min and 500 °C for 1 h and then cooling down to 40 °C, 5% (*v*/*v*) NH_3_/He mixed gas was introduced at a flow rate of 50 mL/min for 1 h for the NH_3_-adsorption step. The physisorbed NH_3_ was then removed by purging with He gas at a flow rate of 50 mL/min for 1 h. The NH_3_-desorption step was performed by heating at a temperature of 500 °C at a heating rate of 10 °C/min. The amount of desorbed NH_3_ was subsequently detected by a TCD. The quantities of weak, medium, and strong acid sites were calculated from the area of the NH_3_–TPD profile according to the temperature to desorb NH_3_ at <200 °C, 200–350 °C, and >350 °C, respectively, [53].

The XPS analysis of the reduced catalysts was conducted in Axis Ultra DLD spectrometer (Kratos, Manchester, UK) equipped with an Al–Kα X-ray source (hν = 1486.6 eV). The reduced catalyst obtained from Section 3.2 was immediately wrapped in aluminum foil and kept in a vacuum plastic bag before analysis. The BE was corrected by taking C 1s (284.8 eV) as the reference energy. The narrow scans of Co 2p and Ti 2p were collected. The XPSPEAK software (version 4.1) was applied in data acquisition using the Shirley method for background subtraction and the Gaussian–Lorentzian function for curve fitting. In addition, the O_v_ was also estimated using the peak area of Ti species (Ti^3+^ and Ti^4+^) detected by XPS analysis using Equation (2) [54].

(2) $$Ov\;(\%)=\frac{\left[\frac{{\mathrm{Area}}_{{\mathrm{Ti}}^{3+}}}{{\mathrm{Area}}_{{\mathrm{Ti}}^{3+}}\;+\;{\mathrm{Area}}_{{\mathrm{Ti}}^{4+}}}\right]}{4}\times 100$$

### 3.4. The HDO of 4PG and Other Lignin-Derived Model Compounds

The catalytic activity of the Co/TiO_2_ catalysts was evaluated in a 20 mL stainless steel autoclave containing the reduced catalyst (150 mg), 4PG (300 mg), *n*-dodecane solvent (4 mL), and a magnetic stirrer bar. For the blank test without the addition of 4PG, the HDO of *n*-dodecane conducted under a 30 bar initial H_2_ pressure and 350 °C showed only 7% *n*-dodecane conversion, indicating a small cracking reaction occurred during the HDO process under a harsh condition. The system was initially purged with H_2_ gas to eliminate the trace oxygen before pressurizing to the desired initial H_2_ pressure. When the reactor reached the reaction temperature, an agitation speed of 700 rpm was applied and maintained for the desired reaction time. After cooling down to room temperature and following the disassembly of the reactor, 4 mL ethyl acetate was added to the product solution to improve the solubility and to dissolve some matters in the product solution [37]. This was followed by 100 µL *n*-octane, used as an internal standard. The spent catalyst and liquid product were separated using centrifugation at 10,000 rpm for 15 min. Other types of lignin-derived model compounds, such as phenol, 4-ally-2-methoxyphenol, and 4-ally-2,6-dimethoxyphenol, were also evaluated using a similar reaction procedure.

To investigate the reusability of the catalysts, the spent Co/TiO_2_ catalysts obtained from the reaction at 310 °C and under a 30 bar initial H_2_ pressure for 1 h were sequentially flushed with ethyl acetate and *n*-dodecane to remove impurities before adding the solution to the vessel with fresh reactant and solvent for a new consecutive run.

To study the effect of the reaction temperature and reaction time, the operating parameters at 280 °C, 30 bar initial H_2_ pressure, and 1 h reaction time were selected as a central condition. The reaction temperature was varied between 190 °C and 310 °C, while the reaction time was adjusted from 0.3 h to 20 h. To assess the reusability of each catalyst, both catalysts were compared under a reaction condition of 310 °C, 30 bar initial H_2_ pressure, and 1 h reaction time, since they showed the same level of 4PG conversion and product distribution in the first run under this condition.

### 3.5. Product Analysis

The liquid product was qualitatively analyzed by GC-MS (Agilent (Santa Clara, CA, USA) 7890A) to identify the liquid products. The liquid product was also analyzed using GC-FID (Agilent 7890A: HP5 column) to evaluate the degree of 4PG conversion and the amount of product species using calibration curves (see Table S6 in Supplementary Materials). For both the GC-FID and GC-MS analyses, the initial column temperature was set to 50 °C before increasing to 280 °C at 10 °C/min with a split ratio of 10 and 75 for the GC-FID and GC-MS, respectively.

The amount of coke deposited on the surface of the spent catalyst (10 mg) was analyzed using TGA (Shimazu (Kyoto, Japan), DTG-60) from room temperature to 800 °C at a heating rate of 10 °C/min under 25 mL/min air flow. Weight loss between room temperature and 200 °C was ascribed to moisture and oxidation of volatile matters. The amount of coke deposited on the surface of the catalyst was then determined from the weight loss between 200 and 800 °C [81]. 

### 3.6. The HDO of Bio-Oil and Product Analysis

The bio-oil used in this study was obtained from the fast pyrolysis of *L. leucocephala* trunk in a cylindrical vibrating reactor (7.32 cm inside diameter, 0.15 cm thickness, and 165 cm length) under an N_2_ atmosphere at a flow rate of 10 mL/min and 500 °C. The HDO of the bio-oil in *n*-dodecane (0.3 g bio-oil/4 mL *n*-dodecane) was conducted following the same procedure reported for 4PG (Section 3.4). However, from the preliminary study, a 1 h reaction time used for the model compounds was not sufficient to eliminate the oxygenated compounds in the real bio-oil, possibly due to the mass transfer limitation caused by the sticky nature of the bio-oil. Thus, a 4 h reaction time was applied to the real bio-oil conversion with variation in the reaction temperature from 310 °C to 350 °C. The gas product was collected after the reactor was cooled to room temperature. Ethyl acetate (8 mL) was applied to rinse the reactor vessel and obtain a homogeneous liquid product. The spent catalyst and liquid product were then separated using centrifugation. 

The composition of the gas product was analyzed by GC-TCD/FID (Shimadzu: GC-2014 using Unibead C column for TCD and Hayasep Q column for FID). The initial column temperature was set to 50 °C for 5 min before heating to 190 °C at 10 °C/min. The content of each compound in the liquid product (as well as bio-oil before the reaction) was reported as the percentage of relative peak area of the GC-MS chromatogram. The water content in the bio-oil and liquid product obtained from the reaction was determined using a volumetric Karl Fischer titrator (Mettler Toledo (Columbus, OH, USA) V20S). In addition, the spent catalyst was evaluated using TGA analysis (PerkinElmer (Shelton, CT, USA), TGA8000) under the same condition as described in Section 3.5 to estimate the solid product deposited on the surface of the catalyst.

## 4. Conclusions

This research investigated the HDO of 4PG and a real bio-oil over two types of Co/TiO_2_ catalysts to enrich the catalyst toolbox for biofuels and bio-based chemicals production. The effect of the TiO_2_ type (TiO_2_–A and TiO_2_–R) on the metal–support interaction and catalytic performance of Co-based catalysts was first evaluated. Although the reaction pathway to convert 4PG was similar for both the Co/TiO_2_–A and Co/TiO_2_–R catalysts, the latter exhibited a higher activity at low temperatures (190–220 °C) and achieved a 100% 4PG conversion level within 20 min. The exceptional performance of the Co/TiO_2_–R catalyst was attributed to its high content of Ti^3+^ species with O_v_ formation during the H_2_ reduction, as detected by XPS and H_2_–TPR analyses. In contrast, the Co/TiO_2_–A catalyst, with its higher acidity, benefited the reactions for a prolonged time to drive the formation of propylcyclohexane. Both catalysts also showed potential for the transformation of other lignin-derived model compounds. However, in terms of reusability, the Co/TiO_2_–A catalyst retained 100% 4PG conversion after four consecutive runs, whereas the Co/TiO_2_–R catalyst was deactivated. When the Co/TiO_2_–A catalyst was used for HDO of the real bio-oil derived from the fast pyrolysis of *Leucaena leucocephala* trunk, it was noticed that the Co/TiO_2_–A produced lower amounts of high oxygenated compounds, such as cellulose derivatives and furan ring compounds. Considering the aromatics, the Co/TiO_2_–A catalyst converted the high oxygenated aromatics (methoxyphenols, dimethoxyphenols, benzenediols, and others) to phenols and, in doing so, enhanced the phenols content from 18.9% in the bio-oil to 61.9% when the HDO was conducted under a 30 bar initial H_2_ pressure at 350 °C for 4 h. This points toward the potential to produce bio-based chemicals derived from LCB conversion in the future with the facile Co/TiO_2_ catalysts.

## Figures and Tables

**Table 1:** Textural properties of TiO_2_ supports and Co/TiO_2_ catalysts.

Sample	Co Amount ^a^	S_BET_^b^	V_p_^c^	r_p_^d^	d_Co_^e^	D_Co_^f^
	(wt%)	(m^2^/g)	(cm^3^/g)	(nm)	(nm)	(%)
TiO_2_-A	-	9.81	0.0156	1.92	-	-
Co/TiO_2_-A	4.92	10.4	0.0173	1.54	19.1	5.02
TiO_2_-R	-	2.92	0.0052	1.93	-	-
Co/TiO_2_-R	4.59	4.19	0.0081	1.53	27.0	3.56

^a^ Determined from the ICP-OES technique. ^b^ S_BET_ = BET surface area. ^c^ V_p_ = total pore volume. ^d^ r_p_ = pore radius. ^e^ d_Co_ = metallic Co crystallite size calculated from the width of XRD peaks (2θ = 44.3°) using Scherrer’s equation [30]. ^f^ D_Co_ = Co dispersion estimated from 6.59S_d_/d_Co_ [30,44].

**Table 2:** H_2_ consumption, percentage reducibility, and acidity of TiO_2_ and Co-based catalysts.

Sample	H_2_ Consumption ^a^	Reducibility ^b^	Acidity ^c^ (µmol NH_3_/g Catalyst)			
	(mmol/g Catalyst)	(%)	Weak	Medium	Strong	Total
TiO_2_-A	-	-	30	9	6	45
Calcined Co/TiO_2_-A	1.06	95.4	40	18	14	72
Reduced Co/TiO_2_-A	-	-	36	9	11	56
TiO_2_-R	-	-	14	10	6	30
Calcined Co/TiO_2_-R	1.17	112.8	17	11	15	46
Reduced Co/TiO_2_-R	-	-	12	7	12	31

^a^ Determined from the H_2_–TPR profiles. ^b^ Calculated from the total amount of H_2_ consumption (obtained from H_2_–TPR analysis) compared to theoretical amount of H_2_ required for Co_3_O_4_ → Co^0^ (based on the Co content from ICP-OES analysis) using the relationship from the Co_3_O_4_ reduction equation reduction [52]. ^c^ Classified as weak (<200 °C), medium (200–350 °C), and strong (350–600 °C) acid sites, as obtained from NH_3_-TPD analysis [53].

**Table 3:** Relative peak area of Co 2p and Ti 2p and the concentration of oxygen vacancies (O_v_) in the calcined and reduced Co/TiO_2_ catalysts, as detected by XPS analysis.

Sample	Peak Area (%)					O_v_^a^ (%)
	Co 2p			Ti 2p		
	Co^0^	CoO_x_	CoO_x_ Satellite	Ti^3+^	Ti^4+^	
Calcined Co/TiO_2_-A	1.72	49.1	49.2	7.83	92.2	1.96
Reduced Co/TiO_2_-A	58.7	32.8	8.50	26.9	73.1	8.22
Calcined Co/TiO_2_-R	1.74	45.3	52.9	11.5	88.5	2.87
Reduced Co/TiO_2_-R	51.4	29.4	19.2	89.8	10.2	22.0

^a^ Concentration of oxygen vacancy O_v_ (%) = $\frac{\left[\frac{{\mathrm{Area}}_{{\mathrm{Ti}}^{3+}}}{{\mathrm{Area}}_{{\mathrm{Ti}}^{3+}}+{\mathrm{Area}}_{{\mathrm{Ti}}^{4+}}}\right]}{4}\times 100$ [54].

## Data Availability

Data are contained within the article and Supplementary Materials.

## Supplementary Materials

The following supporting information can be downloaded at: https://www.mdpi.com/article/10.3390/molecules28227468/s1. Figure S1: representative (a) N_2_ adsorption–desorption isotherms and (b) pore size distribution of the TiO_2_ supports and Co/TiO_2_ catalysts; Figure S2: representative deconvoluted Co 2p XPS spectra of the reduced (a) Co/TiO_2_–A and (b) Co/TiO_2_–R catalysts; Figure S3: representative NH_3_–TPD profiles of the TiO_2_ supports and Co/TiO_2_ catalysts in both calcined and reduced forms; Figure S4: representative TGA profiles of the spent Co/TiO_2_–A and Co/TiO_2_–R catalysts after the fourth consecutive run; Figure S5: distribution of compounds found in the bio-oil before and after HDO over the Co/TiO_2_–A catalyst at different reaction temperatures; Figure S6: representative TGA profiles of the spent Co/TiO_2_–A catalysts obtained from the HDO of bio-oil at different reaction temperatures; Figure S7: representative GC-MS chromatograms of the (a) bio-oil in *n*-dodecane and (b) the liquid product obtained from HDO process using Co/TiO_2_–A catalyst operated under a 30 bar initial H_2_ pressure at 350 °C for 4 h; Table S1: conversion level and product yield from the HDO of several lignin-derived model compounds over Co/TiO_2_ catalysts; Table S2: Co content in the fresh and spent catalysts obtained after the fourth consecutive run; Table S3: types and contents of chemical species found in the bio-oil obtained from the fast pyrolysis of *L. leucocephala* trunk before and after HDO over the Co/TiO_2_–A catalyst (detection by GC-MS analysis); Table S4: gas composition obtained from HDO of dodecane and bio-oil in dodecane; Table S5: compounds in each group of aromatic compounds found in the bio-oil before and after HDO over Co/TiO_2_–A catalyst, as detected by GC-MS analysis; Table S6: calibration data used for calculation of the HDO of all lignin-derived model compounds prepared by GC-FID analysis.
