# Supplementary material for: Hydrodeoxygenation of Oxygenates Derived from Biomass Pyrolysis Using Titanium Dioxide-Supported Cobalt Catalysts
Source: Molecules. 2023 Nov 7;28(22):7468. doi: 10.3390/molecules28227468 (PMC10673104; doi:10.3390/molecules28227468)
Supplement: Supplementary file 1 [file molecules-28-07468-s001.zip › molecules-2660677-supplementary.pdf]

Supplementary files

Hydrodeoxygenation of Oxygenates Derived from Biomass Pyrolysis Using Titanium Dioxide-Supported Cobalt Catalysts

Surachet Hongkailers [1], Adisak Pattiya [2] and Napida Hinchiranan [1,3,4,*]

[1] Department of Chemical Technology, Faculty of Science, Chulalongkorn University, 254 Phyathai Road, Bangkok 10330, Thailand; surachet.hkl@gmail.com
[2] Bio-Energy and Renewable Resources Research Unit, Faculty of Engineering, Mahasarakham University, Kamriang, Kantharawichai, Maha Sarakham 44150, Thailand; adisak_pattiya@yahoo.com
[3] Center of Excellence on Petrochemical and Materials Technology (PETROMAT), Chulalongkorn University, 254 Phyathai Road, Bangkok 10330, Thailand
[4] Center of Excellence in Catalysis for Bioenergy and Renewable Chemicals (CBRC), Chulalongkorn University, 254 Phyathai Road, Bangkok 10330, Thailand
* Correspondence: napida.h@chula.ac.th

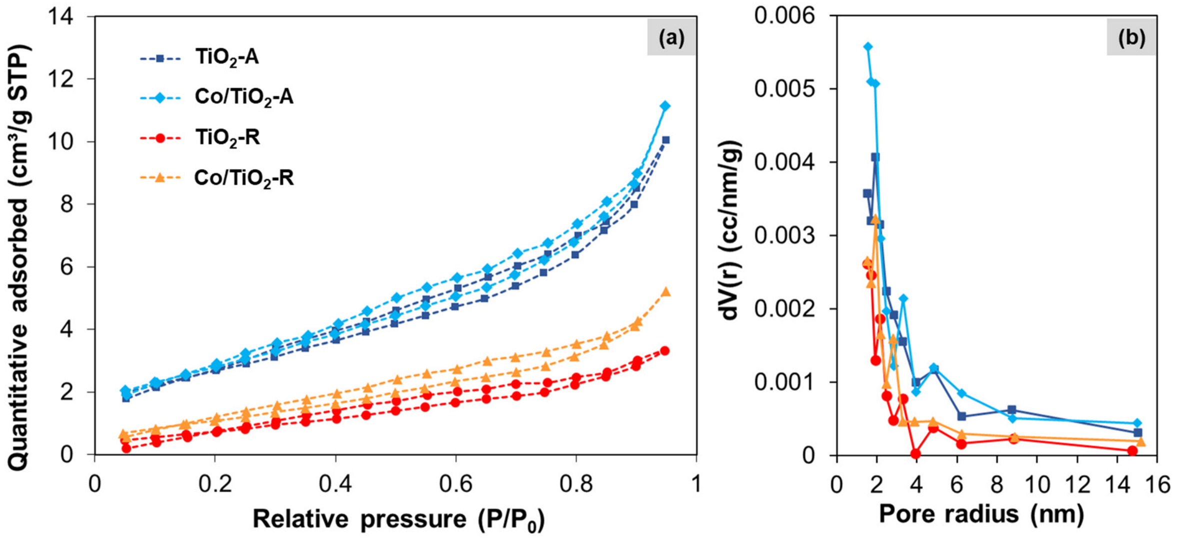

Figure S1. (a) N_2 adsorption-desorption isotherms and (b) pore size distribution of the TiO_2 supports and Co/TiO_2 catalysts.

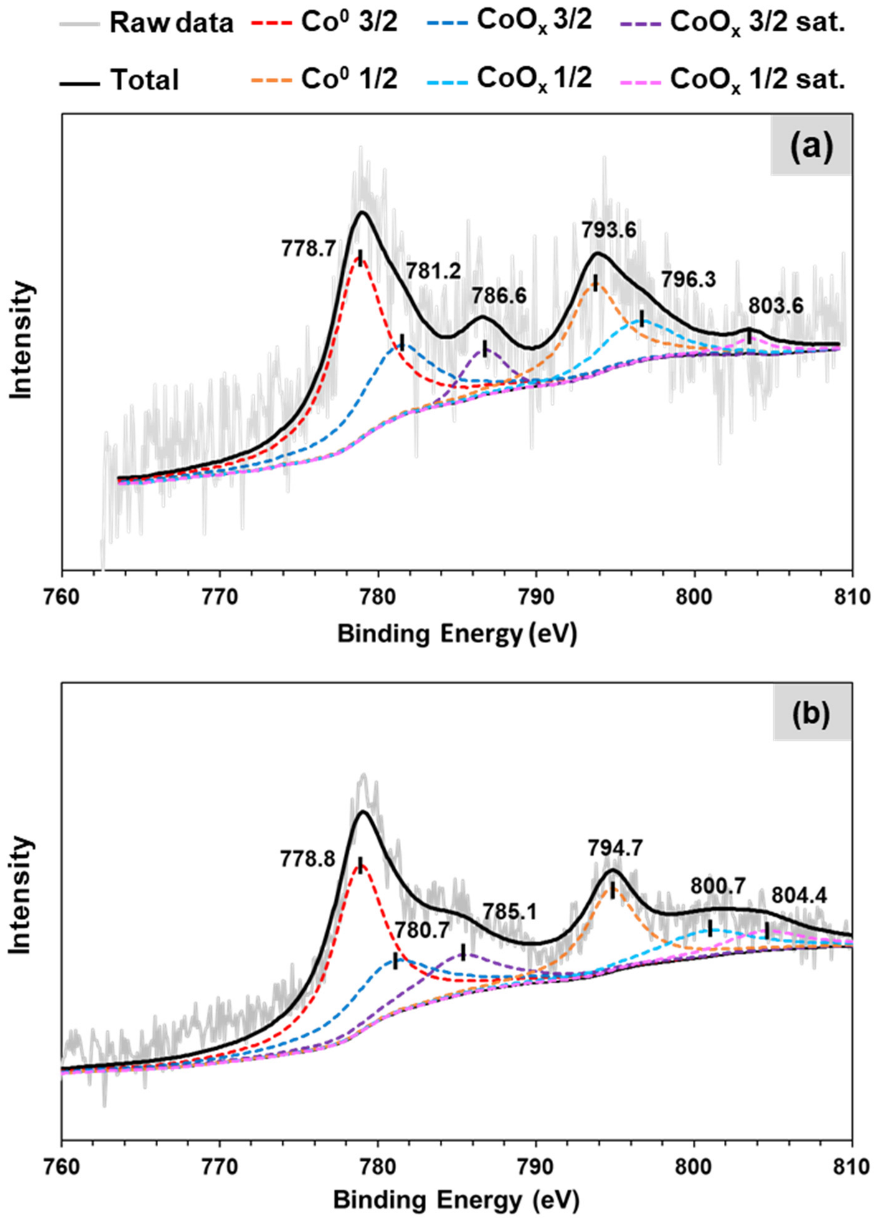

Figure S2. Representative deconvoluted Co 2p XPS spectra of the reduced (a) Co/TiO_2-A and (b) Co/TiO_2-R catalysts.

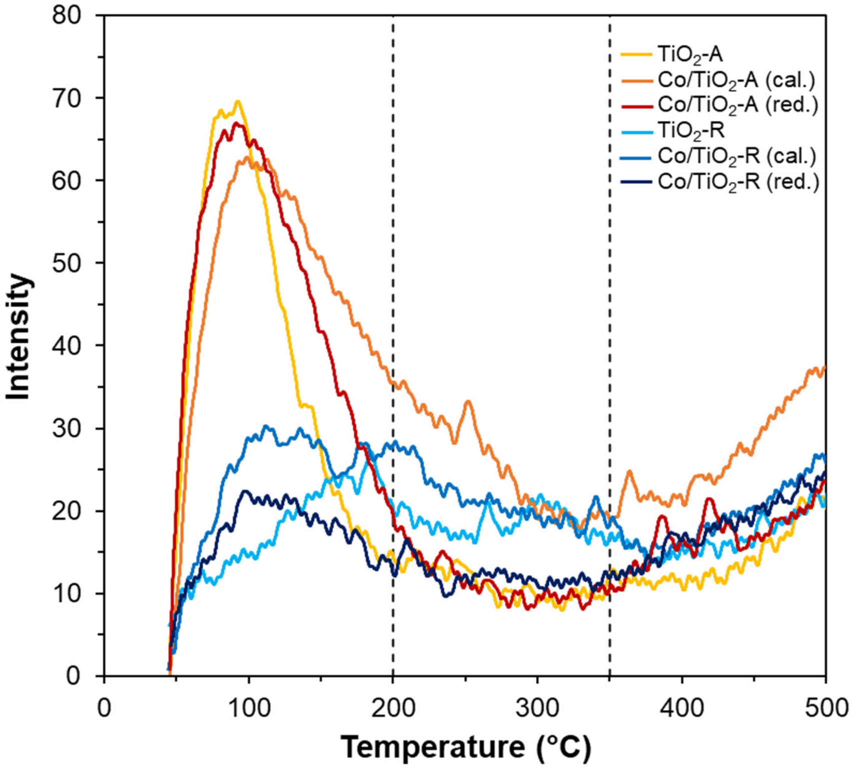

Figure S3. Representative NH_3-TPD profiles of the TiO_2 supports and Co/TiO_2 catalysts in both calcined and reduced forms.

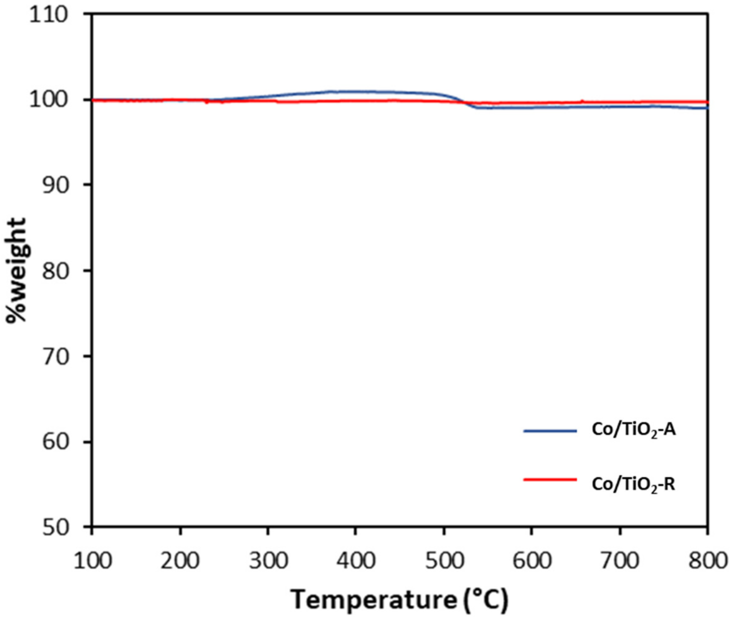

Figure S4. Representative TGA profiles of the spent Co/TiO_2-A and Co/TiO_2-R catalysts after the fourth consecutive run.

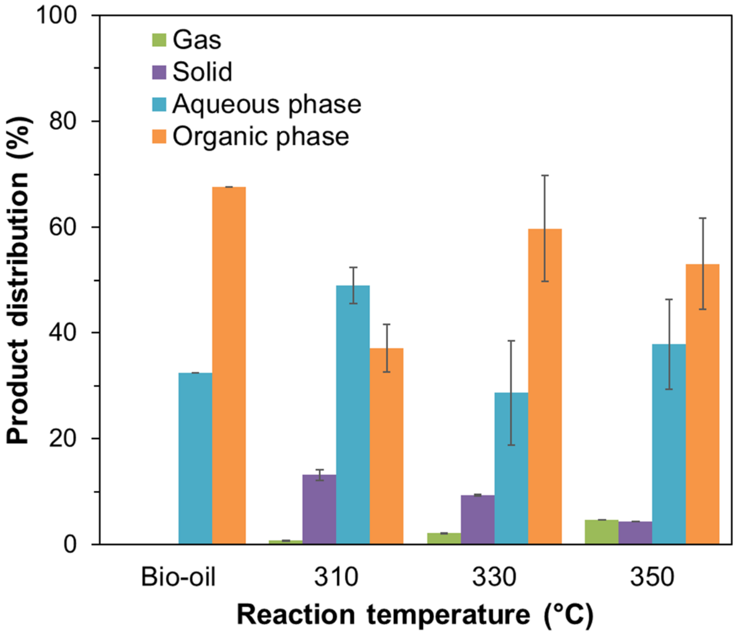

Figure S5. Distribution of compounds found in the bio-oil before and after HDO over the Co/TiO_2-A catalyst at different reaction temperatures (Condition: 30 bar initial H_2 pressure and 4 h).

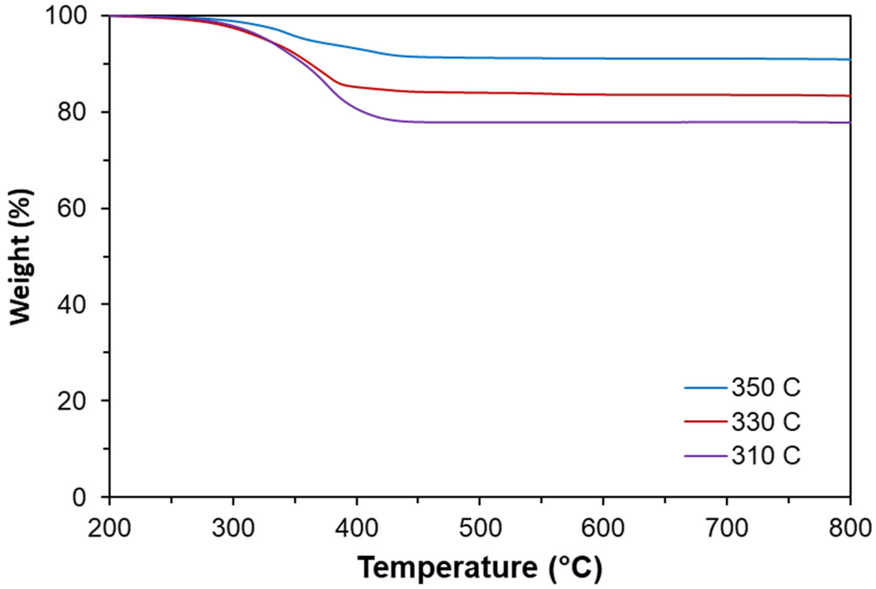

Figure S6. Representative TGA profiles of the spent Co/TiO_2-A catalysts obtained from the HDO of bio-oil at different reaction temperatures (Condition: 30 bar initial H_2 pressure and 4 h).

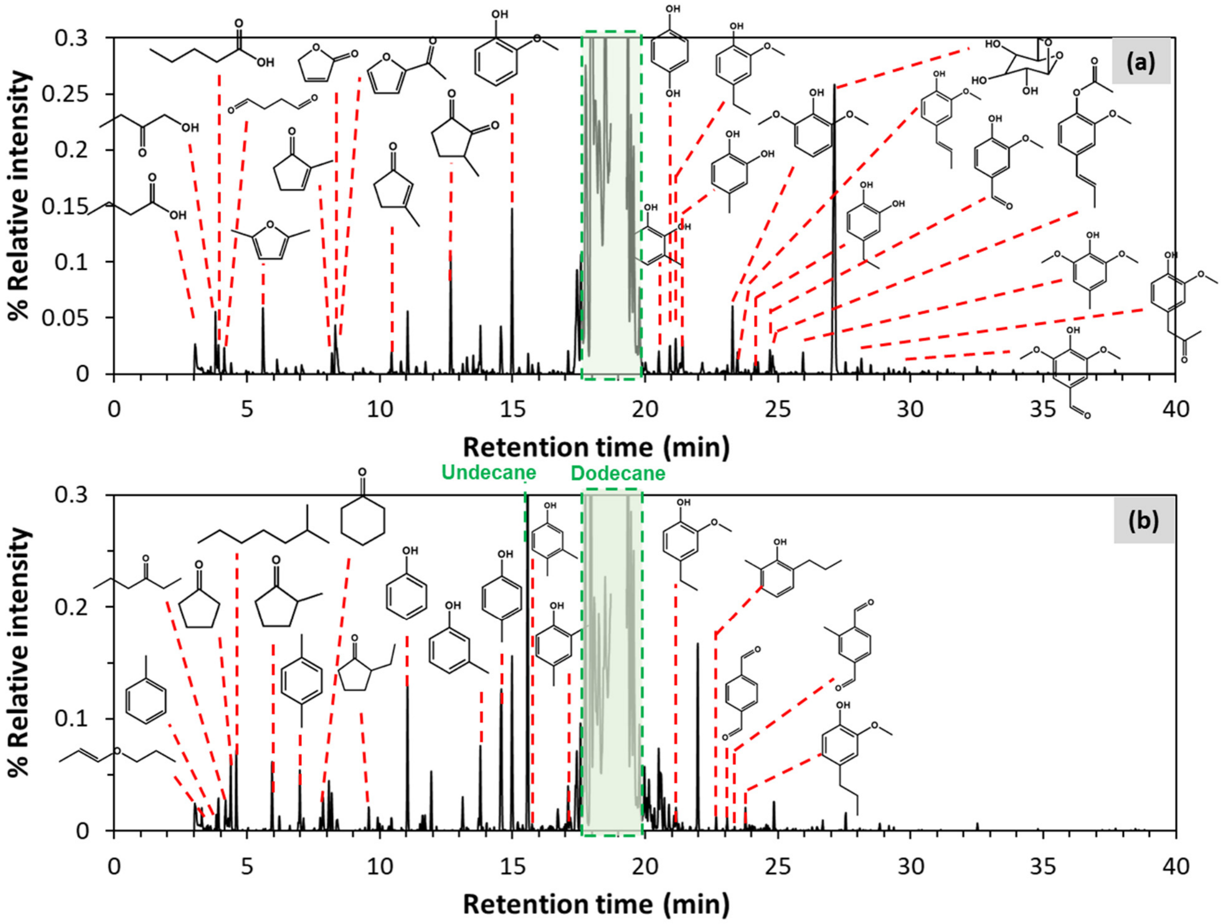

Figure S7. Representative GC-MS chromatograms of the (a) bio-oil in n-dodecane and (b) the liquid product obtained from HDO process using Co/TiO_2-A catalyst operated under 30 initial H_2 pressure at 350 °C for 4 h.

Table S1. Conversion level and product yield from the HDO of several lignin-derived model compounds over Co/TiO_2 catalysts.

Substrate	Catalyst	Conversion (%)	Product yield (%)				
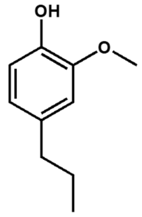 4PG	Co/TiO_2-A	99.8	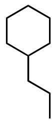 48.3	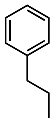 3.0	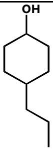 40.5	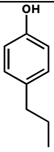 0.8	
	Co/TiO_2-R	99.9	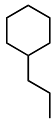 43.4	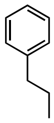 6.8	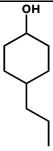 41.1	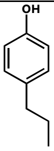 0.5	
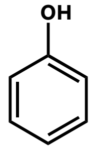 Phenol	Co/TiO_2-A	99.7	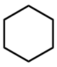 18.0	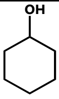 82.0			
	Co/TiO_2-R	100	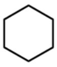 23.9	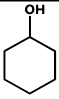 76.1			
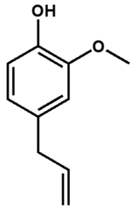 4-Ally-2-methoxyphenol	Co/TiO_2-A	100	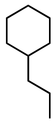 16.4	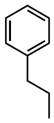 2.0	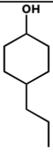 73.0	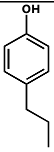 0.3	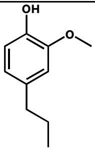 1.0
	Co/TiO_2-R	100	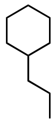 16.1	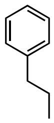 2.2	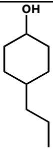 69.6	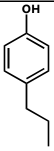 0.3	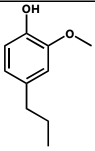 0.8
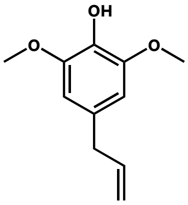 4-Ally-2,6-dimethoxyphenol	Co/TiO_2-A	99.0	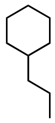 6.8	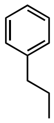 1.8	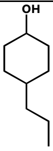 59.0	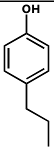 2.5	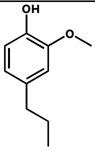 1.7
	Co/TiO_2-R	99.8	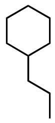 23.7	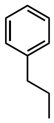 6.2	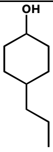 48.5	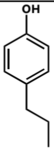 1.8	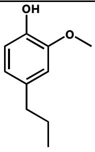 1.1

Condition: 30 bar initial H_2 pressure at 310 °C for 1 h using 300 mg substrate, 150 mg catalyst, and 4 mL n-dodecane

Table S2. The Co content in the fresh and spent catalysts obtained after the fourth consecutive run.

Sample	Co content (wt%)	
	Fresh catalyst	Spent catalyst
Co/TiO_2-A	4.92	4.55
Co/TiO_2-R	4.59	2.20

Table S3. Types and contents of chemical species found in the bio-oil obtained from the fast pyrolysis of *L. leucocephala* trunk before and after HDO over the Co/TiO_2-A catalyst (detection by GC-MS analysis).

RT (min)	Component	Relative peak area (%)	
		Bio-oil	Hydrotreated bio-oil[a]
**Non-oxygenated aliphatics**			
4.60	i-Octane	n.d.	4.82
7.75	1,2-Dimethylcyclopentane	n.d.	0.76
11.61	3,5-Dimethyl-1-hexene	n.d.	0.93
**Oxygenated aliphatics**			
3.28	Butanoic acid	1.09	n.d.
3.29	1-Propenyl propyl ether	n.d.	2.22
3.81	1-Hydroxy-2-butanone	3.02	n.d.
3.92	Pentanoic acid	1.46	2.35
4.14	Butanedial	1.29	n.d.
4.19	3-Hexanone	n.d.	2.56
4.30	2-Hexanone	n.d.	1.00
4.40	Cyclopentanone	0.53	4.77
5.94	2-Methylcyclopentanone	n.d.	4.35
6.22	3-Methylcyclopentanone	n.d.	0.91
7.13	Hexanoic acid	n.d.	0.91
7.86	Cyclohexanone	n.d.	2.61
8.20	2-Methyl-2-cyclopentenone	1.15	2.61
9.44	2-Methyl-4-hexene-3-one	n.d.	0.20
9.59	2-Ethylcyclopentanone	n.d.	1.43
9.92	2-Methylcyclohexanone	n.d.	0.47
10.44	3-Methyl-2-cyclopentenone	1.18	0.92
11.51	3,5-Heptadien-2-one	n.d.	0.39
11.72	3-Methylcyclohexnene-1-one	0.63	0.93
12.67	3-Methyl-1,2-cyclopentanedione	6.20	n.d.
13.12	2,3-Dimethyl-2-cyclopenten-1-one	0.37	2.23
13.52	2,2-Dimethyl-3-heptanone	0.86	n.d.
13.87	3-Methylcyclohexanone	n.d.	0.79
15.73	3-Hydroxy-2-methyl-4H-Pyran-4-one	0.44	n.d.
22.93	Isobutyric acid anhydride	0.03	n.d.
24.25	1,1-Dimethylpropyl 2-ethylhexanoate	0.61	n.d.
**Non-oxygenated aromatics**			
3.84	Toluene	n.d.	0.80
6.99	p-Xylene	n.d.	3.54

Table S3. (Continued)

RT (min)	Component	Content (mmol/$g_{bio\text{-}oil}$)	
		**Bio-oil**	**Hydrotreated bio-oil[a]**
**Oxygenated aromatics**			
11.04	Phenol	3.19	9.79
13.79	3-Methylphenol	2.11	5.47
14.55	4-Methylphenol	3.52	14.41
14.98	2-Methoxyphenol	9.09	12.75
15.72	3,4-Dimethylphenol	n.d.	0.36
17.09	2,4-Dimethylphenol	1.20	3.04
17.17	3,5-Dimethylphenol	n.d.	1.29
17.67	4-(2-Hydroxyethyl)phenol	0.50	2.62
20.52	3-Methyl-1,2-benzenediol	1.22	n.d.
20.94	1,4-Benzenediol	1.87	n.d.
21.15	4-Ethyl-2-methoxyphenol	2.18	1.68
21.26	1,3-Benzenediol	n.d.	0.35
21.41	4-Methyl-1,2-benzenediol	2.28	0.29
22.67	2-Methyl-6-propylphenol	n.d.	0.86
23.09	1,4-Benzenedicarboxaldehyde	n.d.	0.85
23.10	5-Methyl-1,3-benzenediol	0.41	n.d.
23.30	2,6-Dimethoxylphenol	3.66	n.d.
23.37	2-Methyl-1,4-benzenedicarboxaldehyde	n.d.	0.25
23.48	4-(1-Propenyl)-2-methoxyphenol	1.18	n.d.
23.78	4-Propylguaiacol	n.d.	1.30
24.13	4-Ethyl-1,2-benzenediol	0.55	n.d.
24.70	4-Hydroxy-3-methoxybenzaldehyde	1.39	n.d.
24.94	2-Methoxy-4-propenylphenyl acetate	0.07	n.d.
25.95	4-Methyl-2,6-dimethoxyphenol	1.05	n.d.
28.15	4-Hydroxy-3-methoxyphenyl acetone	0.81	n.d.
29.78	4-Hydroxy-3,5-dimethoxybenzaldehyde	0.34	n.d.
**Furan-ring compounds**			
5.60	2,5-Dimethylfuran	3.81	n.d.
8.32	2(5H)-Furanone	2.85	n.d.
8.38	Acetylfuran	1.73	1.27
9.37	5-Methyl-2(5H)-furanone	0.31	n.d.
10.38	5-Methylfurfural	0.14	0.12
10.79	3-Methyl-2(5H)-furanone	0.75	n.d.
11.37	3-Hydroxydihydro-2(3H)-furanone	0.58	n.d.
13.28	5-Hydroxymethyl-2(5H)-furanone	0.88	n.d.

Table S3. (Continued)

RT (min)	Component	Content ($mmol/g_{bio-oil}$)	
		**Bio-oil**	**Hydrotreated bio-oil[a]**
14.02	Tetrahydrofurfuryl alcohol	n.d.	0.46
15.42	Ethylfuran	n.d.	0.33
28.50	3,4-Dihydroxy-5-methyl-dihydrofuran-2-one	0.40	n.d.
**Cellulose derivative compounds**			
27.14	1,6-Anhydro-β-D-glucopyranonse (levoglucosan)	27.19	n.d.
**Total**		**100.00**	**100.00**

n.d. = could not be detected
[a] Obtained from HDO process operated at 350 °C under 30 bar initial H_2 pressure for 4 h

Table S4. Gas composition obtained from HDO of dodecane and bio-oil in dodecane.

Condition[a]	Gas concentration (mol%)					
	C_1	C_2	C_3	C_4	CO	CO_2
Bio-oil in dodecane[b] at 310 °C	0.07	0.02	0.03	0.01	0.05	0.17
Bio-oil in dodecane[b] at 330 °C	0.10	0.04	0.03	0.01	0.09	0.56
Bio-oil in dodecane[b] at 350 °C	0.26	0.19	0.08	0.01	0.22	1.29
Dodecane[c] at 350 °C	0.07	0.04	0.01	0.00	0.00	0.00

[a]Reaction condition: 30 bar initial H_2 pressure, 4 h.; [b]Bio-oil/dodecane = 0.3 g/4 mL; [c]dodecane = 4 mL

Table S5. Compounds in each group of aromatic compounds found in the bio-oil before and after HDO over Co/TiO_2-A catalyst, as detected by GC-MS analysis.

Chemical
**Non-oxygenated arenes**
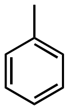 toluene
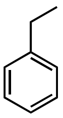 ethylbenzene
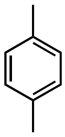 p-xylene
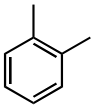 o-xylene
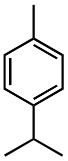 1-methyl-3-isopropylbenzene
**Phenols**
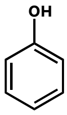 phenol
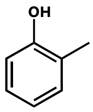 2-methylphenol
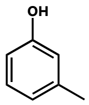 3-methylphenol
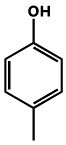 4-methylphenol
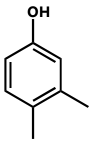 3,4-dimethylphenol
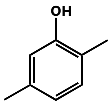 2,5-dimethylphenol
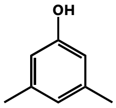 3,5-dimethylphenol
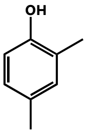 2,4-dimethylphenol
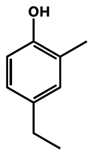 4-ethyl-2-methylphenol
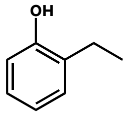 2-ethylphenol
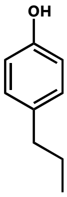 4-propylphenol
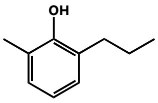 2-methyl-6-propylphenol
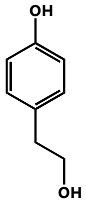 4-(2-hydroxyethyl)phenol
**Methoxyphenols**
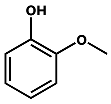 2-methoxyphenol
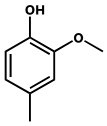 4-methyl-2-methoxyphenol
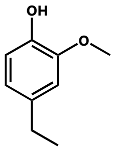 4-ethyl-2-methoxyphenol
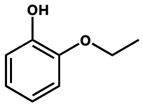 2-ethoxyphenol
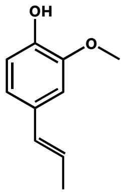 4-(1-propenyl)-2-methoxyphenol
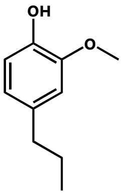 4-propyl-2-methoxyphenol
**Dimethoxyphenols**
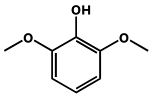 2,6-dimethoxyphenol
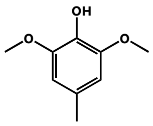 4-methyl-2,6-dimethoxyphenol

Table S5. (continued)

Chemical						
**Benzenediols**						
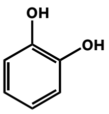	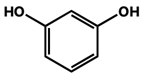	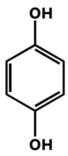	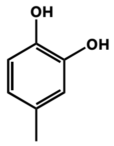	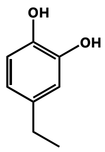	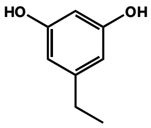	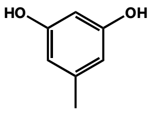
1,2-benzenediol	1,3-benzenediol	1,4-benzenediol	4-methyl-1,2-benzenediol	4-ethyl-1,2-benzenediol	5-ethyl-1,3-benzenediol	5-methyl-1,3-benzenediol
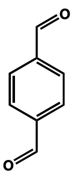	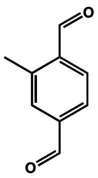					
1,4-benzendicarboxaldehyde	2-methyl-1,4-benzendicarboxaldehyde					
**Others**						
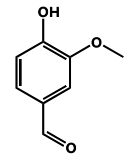	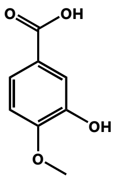	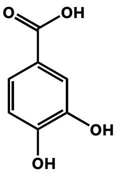	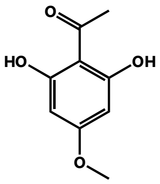	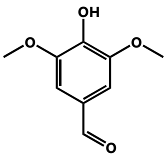	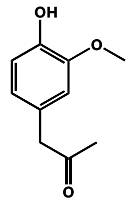	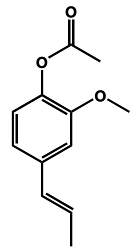
4-hydroxy-3-methoxybenzaldehyde	3-hydroxy-4-methoxybenzoic acid	3,4-dihydroxybenzoic acid	2,6-dihydroxy-4-methoxyacetophenone	4-hydroxy-3,5-dimethoxybenzaldehyde	4-hydroxy-3-methoxyphenyl acetone	2-methoxy-4-(1-propenyl)phenyl acetate

Table S6. Calibration data used for calculation of the HDO of all lignin-derived model compounds prepared by GC-FID analysis

Component	Equation	R^2
4-Propylguaiacol	y = 0.9431x - 0.0048	0.9999
4-Propylphenol	y = 0.8971x - 0.0064	0.9998
Propylbenzene	y = 1.0499x - 0.0102	0.9999
Propylcyclohexane	y = 1.0589x + 0.0074	0.9999
Cyclohexane	y = 0.789x - 0.0201	0.9998
Cyclohexanol	y = 0.6076x - 0.0006	0.9999
Eugenol	y = 0.8971 - 0.0064	0.9998
4-Ally-2,6-dimethoxyphenol	y = 0.7851x - 0.0085	0.9996

y = peak area of product (i)/peak area of internal standard;
x = mol of product (i)/mol of internal standard
